# Trends in Use of Oral Anticoagulants in Older Adults With Newly Diagnosed Atrial Fibrillation, 2010-2020

**DOI:** 10.1001/jamanetworkopen.2022.42964

**Published:** 2022-11-18

**Authors:** Darae Ko, Kueiyu Joshua Lin, Lily G. Bessette, Su Been Lee, Allan J. Walkey, Susan Cheng, Erin Kim, Robert J. Glynn, Dae Hyun Kim

**Affiliations:** 1 Section of Cardiovascular Medicine, Boston University School of Medicine, Boston, Massachusetts; 2 Division of Pharmacoepidemiology and Pharmacoeconomics, Department of Medicine, Brigham and Women’s Hospital, Boston, Massachusetts; 3 Division of General Internal Medicine, Department of Medicine, Massachusetts General Hospital, Harvard Medical School, Boston, Massachusetts; 4 The Pulmonary Center, Boston University School of Medicine, Boston, Massachusetts; 5 Department of Cardiology, Cedars-Sinai Medical Center, Los Angeles, California; 6 Hinda and Arthur Marcus Institute for Aging Research, Hebrew Senior Life, Boston, Massachusetts

## Abstract

**Importance:**

Undertreatment of older adults with atrial fibrillation with anticoagulation therapy is an important practice gap. It has been posited that the availability of direct oral anticoagulants (DOACs) would improve oral anticoagulant (OAC) initiation in older adults with atrial fibrillation given their superior safety profile compared with warfarin.

**Objectives:**

To systematically examine trends in OAC initiation and nonadherence in older adults with atrial fibrillation and coexisting geriatric conditions.

**Design, Setting, and Participants:**

This retrospective cohort study uses administrative claims data from Optum’s Clinformatics Data Mart from January 1, 2010, to December 31, 2020. Participants included beneficiaries of Medicare Advantage plans aged 65 years and older with atrial fibrillation and elevated risk of ischemic stroke. Data analysis was performed from October 2021 to October 2022.

**Exposures:**

Coexisting dementia, frailty, and anemia.

**Main Outcomes and Measures:**

The primary outcomes were OAC initiation within 12 months after the first diagnosis of atrial fibrillation per year and nonadherence with OAC per year (defined as <80% of proportion of days covered among patients newly started on OAC in each year).

**Results:**

There were 21 603 to 51 236 patients per year (total for 2010-2020, 381 488 patients) in the OAC-eligible incident AF cohort (mean [SD] age, 77.2 [6.1] to 77.4 [6.8] years; 13 871 [51.8%] to 22 901 [49.8%] women). OAC initiation within 12 months after incident AF increased from 20.2% (5405 of 26 782 patients) in 2010 to 32.9% (7111 of 21 603 patients) in 2020. DOAC uptake increased from 1.1% (291 of 26 782 patients) to 30.9% (6678 of 21 603 patients), and warfarin initiation decreased from 19.1% (5114 of 26 782 patients) to 2.0% (436 of 21 603 patients). Older age (odds ratio [OR], 0.98; 95% CI, 0.98-0.98), dementia (OR, 0.57; 95% CI, 0.55-0.58), frailty (OR, 0.74; 95% CI, 0.72-0.76), and anemia (OR, 0.75; 95% CI, 0.74-0.77) were associated with lower odds of OAC initiation. During the study period, the median (IQR) proportion of days covered increased from 77.6% (41.0%-96.4%) to 90.2% (57.4%-98.6%), and OAC nonadherence decreased from 52.2% (2290 of 4389 patients) to 39.0% (3434 of 8798 patients).

**Conclusions and Relevance:**

Since the introduction of DOACs, OAC initiation in older adults with has improved but remained suboptimal in 2020. Additional strategies are needed to improve stroke prophylaxis in all older adults with atrial fibrillation including those with coexisting dementia, frailty, and anemia.

## Key Points

**Question:**

Did timely initiation of and adherence to oral anticoagulants change between 2010 and 2020 among anticoagulation-eligible older adults with atrial fibrillation (AF)?

**Findings:**

In this cohort study of 381 488 Medicare beneficiaries with incident AF between 2010 and 2020, oral anticoagulant initiation within 12 months of new AF diagnosis improved from 20.2% to 32.9%, and nonadherence decreased from 52.2% to 39.0%. Patients with dementia, frailty, and anemia were less likely than patients without those conditions to initiate an oral anticoagulant.

**Meaning:**

These findings suggest that stroke prophylaxis using oral anticoagulation for AF has improved in the past decade, but older adults with AF and coexisting dementia, frailty, and anemia remain undertreated.

## Teaser

This cohort study examines trends in oral anticoagulant initiation and nonadherence in older adults with atrial fibrillation and coexisting geriatric conditions.

## Introduction

The prevalence of atrial fibrillation (AF) increases exponentially with advancing age,^1^ and cardioembolic stroke resulting from AF accounts for 1 in every 3 ischemic strokes in adults older than 65 years.^2^ AF increases the risk of cardioembolic stroke 5-fold without anticoagulation,^3^ and AF-related strokes are more often fatal and cause greater disability compared with non-AF strokes.^4,5^ Warfarin reduces risk of ischemic stroke and systemic embolism by 64% compared with placebo and by 37% compared with antiplatelets,^6^ but only approximately 55% of older adults with AF and guideline eligibility for an oral anticoagulant (OAC) were prescribed warfarin in 2010.^7^ Bleeding risk, fall risk, frailty, cognitive impairment, and complexity of adherence to warfarin were the commonly cited reasons for warfarin noninitiation^8,9^ and discontinuation.^10^

It has been posited that the availability of direct OACs (DOACs) would substantially improve OAC initiation in older adults with AF given their superior safety profile^11^ and relative ease of use compared with warfarin. Indeed, among Medicare fee-for-service (FFS) beneficiaries aged 65 years and older, OAC initiation any time after a new AF diagnosis increased from 34% in 2011 to 53% in 2016 in parallel with DOAC uptake.^12^ The changes in OAC initiation and adherence after 2016 in the US among a population at highest risk for noninitiation and nonadherence have not been systematically studied.

Understanding contemporary OAC prescription patterns and identification of older patients who stand to benefit the most from OAC but remain untreated will enable the design of implementation strategies to improve evidence-based care. In the current study, using a US national administrative claims database, we aimed to examine (1) trends in OAC initiation and DOAC uptake from 2010 to 2020 among older adults with new AF at elevated risk of stroke, (2) patient characteristics associated with noninitiation of OAC and DOAC after new AF diagnosis, and (3) trends in OAC nonadherence.

## Methods

This cohort study was approved by the institutional review board at Brigham and Women’s Hospital, Boston, Massachusetts, and a waiver of informed consent was obtained because the data were deidentified, in accordance with 45 CFR §46. The Strengthening the Reporting of Observational Studies in Epidemiology (STROBE) reporting guideline for cohort studies was followed.

### Database

We analyzed administrative claims data between January 1, 2010, and December 31, 2020, from Optum’s Clinformatics Data Mart to identify Medicare Advantage (MA) plan beneficiaries aged 65 years or older with AF. During our study period, the proportion of Medicare beneficiaries enrolled in MA plans has been increasing, from 24% in 2010 to 42% in 2020.^13^ This database includes deidentified information on diagnoses and procedures, pharmacy, and use of health care services.

### Study Cohorts

**Figure 1.**
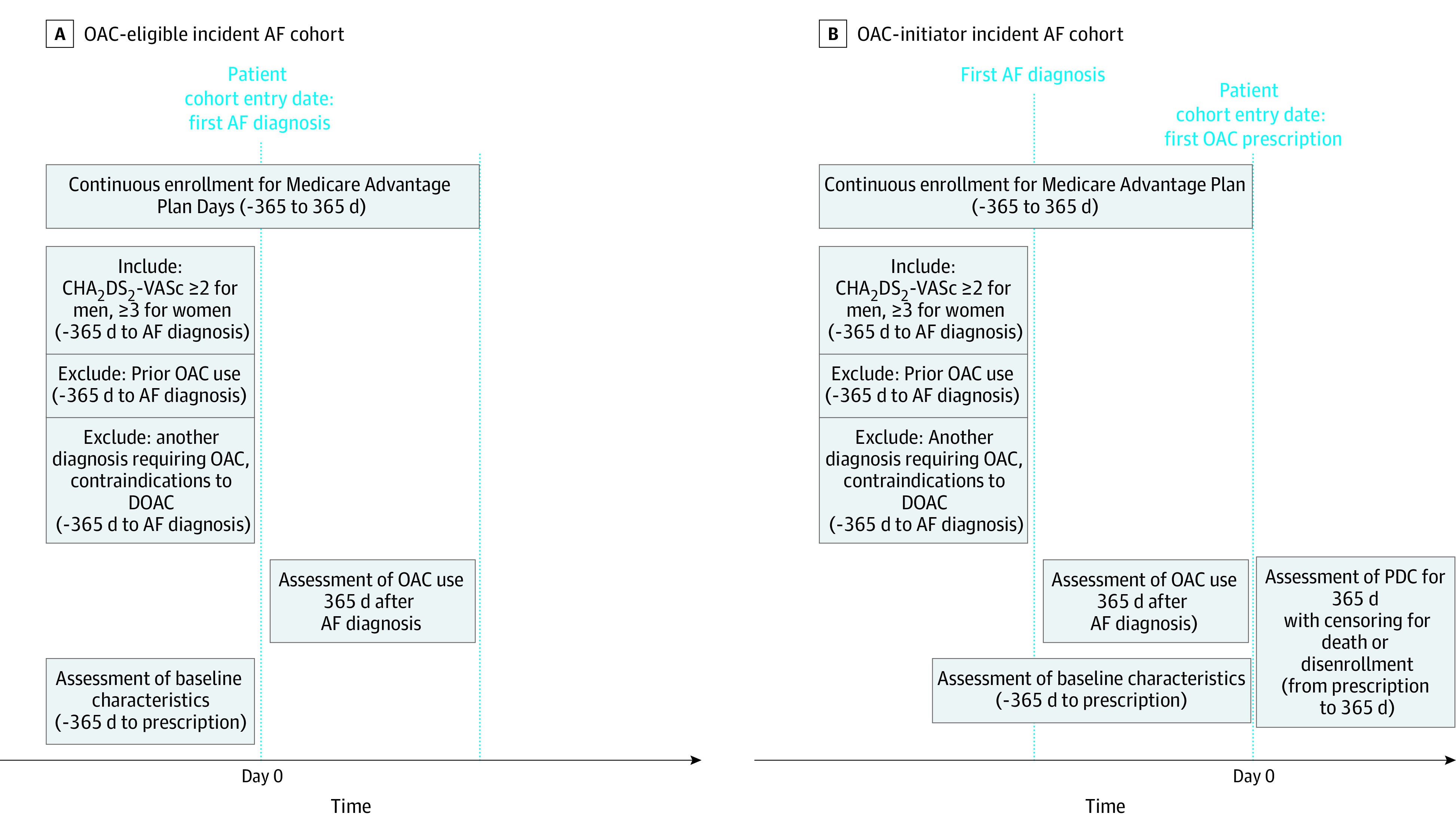
Study Cohort Definitions A, We designed the oral anticoagulant (OAC)–eligible incident atrial fibrillation (AF) cohort to examine trends in 12-month initiation of OAC and direct oral anticoagulant (DOAC) uptake and to identify patient characteristics associated with noninitiation of OAC and DOAC. B, The OAC-initiator incident AF cohort was designed to examine trends in OAC nonadherence. CHA_2_DS_2_-VASc indicates 1 point for congestive heart failure, 1 point for hypertension, 2 points for age 75 years or older, 1 point for age 65 to 74 years, 1 point for diabetes, 2 points for history of stroke or transient ischemic attack or systemic thromboembolism, 1 point for vascular disease including myocardial infarction or peripheral arterial disease, and 1 point for female sex; PDC, proportion of days covered.

We defined 2 cohorts to examine trends in OAC and DOAC initiation, identify patient characteristics associated with noninitiation of OAC and DOAC (OAC-eligible incident AF cohort), and to examine trends in OAC nonadherence (OAC-initiator incident AF cohort). The OAC-eligible incident AF cohort included patients who received a first AF diagnosis and who met all of the following criteria in the 365 days before and on the cohort entry date (Figure 1A): (1) 1 or more inpatient or 2 or more outpatient diagnoses of AF, (2) CHA_2_DS_2_-VASc (1 point for congestive heart failure, 1 point for hypertension, 2 points for age ≥75 years, 1 point for age 65-74 years, 1 point for diabetes, 2 points for history of stroke or transient ischemic attack or systemic thromboembolism, 1 point for vascular disease including myocardial infarction or peripheral arterial disease, and 1 point for female sex) score 2 or more points for men and 3 or more points for women, (3) no other indications for OAC (mechanical valves or venous thromboembolism), and (4) no contraindications to DOAC and warfarin (mitral stenosis, rheumatic heart disease, end-stage renal disease, or intracranial hemorrhage).^14^ To measure medical comorbidities and drug exposure, we restricted our analysis to those who were continuously insured in the year before and after the cohort entry date. The cohort entry date was defined as the day of first inpatient AF diagnosis or first of 2 outpatient AF diagnoses occurring within 90 days of each other. To examine OAC initiation within 12 months of the AF diagnosis, we also excluded patients who had any OAC prescription in the 365 days before the cohort entry date. The OAC-initiator incident AF cohort was a subset of patients in the OAC-eligible incident AF cohort who received their prescription for warfarin or DOAC within 365 days after the AF diagnosis. The cohort entry date was defined as the day of the first OAC prescription (Figure 1B).

### Measurements of OAC Initiation and Nonadherence

OAC initiation was defined as filling 1 or more prescription for warfarin, dabigatran (approved October 19, 2010), rivaroxaban (approved November 4, 2011), or apixaban (approved December 28, 2012) for the first time within 12 months of a new AF diagnosis. We did not include edoxaban (approved January 8, 2015) because it constituted 0.01% to 0.02% (54 patients) of all OAC use. Patients were assigned to the first drug that was filled. Among the patients with first AF diagnosis who were initiated on OAC, we calculated the proportion of days covered (PDC) during a 1-year period from the date of first OAC prescription. The Centers for Medicare & Medicaid Services use PDC to evaluate the quality of its prescription coverage plans and uses PDC less than 80% to define nonadherence.^15^

### Measurements of Patient Characteristics

Using the look-back period of 365 days including the cohort entry date, we captured age, sex, self-reported race and ethnicity (Asian, Black, Hispanic, White, and other [American Indian, Pacific Islander, unknown, and any other race]), and chronic conditions, including anemia, chronic kidney disease, dementia, hip or pelvic fracture, stroke, and transient ischemic attack (TIA), according to the claims-based algorithms listed by the Centers for Medicare & Medicaid Services Chronic Conditions Data Warehouse.^16^ Race and ethnicity were assessed in this study because anticoagulation utilization was previously shown to be different by race and ethnicity.^17^ The comorbidity burden was quantified using the Combined Comorbidity Index.^18^ Frailty was measured using a validated claims-based frailty index,^19,20,21,22^ which estimates a deficit-accumulation frailty index (range, 0-1) using 93 variables defined by diagnosis, health services, and durable medical equipment codes. Patients with claims-based frailty index greater than or equal to 0.25 were classified as being frail. Baseline stroke risk was quantified using CHA_2_DS_2_-VASc score, which was categorized as lower risk (≤2 for men and ≤3 for women) or higher risk (>2 for men and >3 for women). Baseline bleeding risk was quantified using the HAS-BLED score (1 point each for hypertension, renal disease, liver disease, prior stroke, history of bleeding, age >65 years, use of aspirin and other antiplatelets, and alcohol use disorder, excluding labile international normalized ratio),^23^ which was categorized as lower (<4) or higher risk (≥4). We excluded the labile international normalized ratio component because it cannot be applied to DOACs.^24^

### Statistical Analysis

We examined trends of OAC initiation and DOAC uptake from 2010 to 2020 using the OAC-eligible incident AF cohort and tested the time trends using logistic regression with calendar year as the independent variable adjusting for temporal change in patients’ demographic characteristics and comorbidities. For each calendar year, we calculated the proportion of OAC-eligible patients with incident AF who filled warfarin, dabigatran, rivaroxaban, or apixaban prescriptions within 1 year after the AF diagnosis. We conducted subgroup analyses by age (65-79 vs ≥80 years), sex, race and ethnicity (White, Black, Hispanic, Asian, and other [American Indian, Pacific Islander, any other race, and unknown]), presence of frailty, dementia, prior stroke, chronic kidney disease, CHA_2_DS_2_-VASc score (higher risk vs lower risk), and HAS-BLED score (higher risk vs lower risk). To identify patient characteristics associated with noninitiation of OAC and DOACs, we used multivariable logistic regression to assess the association of clinical characteristics with (1) any OAC use (vs no use) and (2) DOAC use (vs warfarin use) in the OAC-eligible incident AF cohort. Finally, we calculated the median (IQR) of PDC and the proportion of warfarin or DOAC users who were nonadherent (ie, PDC <80%) during the 1-year period after OAC initiation by calendar year. We assessed nonadherence in the subgroups defined already. Analyses were conducted in the Aetion Evidence Generation Platform version 4.6.1^25^ (including R statistical software version 3.4.2; R Project for Statistical Computing). A 2-sided *P* < .05 was considered statistically significant. Data analysis was performed from October 2021 to October 2022.

## Results

### Characteristics of Study Population

The OAC-eligible incident AF cohort ranged from 21 603 to 51 236 patients per year (total for 2010-2020, 381 488 patients) (Table 1). The sample size decreased in 2020 when the COVID-19 pandemic started. The mean (SD) age of the cohort was 77.2 (6.1) to 77.4 (6.8) years; 13 871 (51.8%) to 22 901 (49.8%) patients were women. The proportion of White individuals decreased from 78.4% (21 004 of 26 782 individuals) in 2010 to 74.7% (16 131 of 21 603 individuals) in 2020; the proportion of Black individuals increased from 7.4% (1986 of 26 782 individuals) to 8.8% (1908 of 21 603 individuals), that of Hispanic individuals increased from 7.5% (2022 of 26 782 individuals) to 8.8% (1900 of 21 603 individuals), and that of individuals of other races increased from 4.5% (1212 of 26 782 individuals) to 5.6% (1205 of 21 603 individuals). The overall burden of comorbidities was high, with the overall mean (SD) Combined Comorbidity Index increasing from 2.7 (2.3) in 2010 to 3.7 (2.8) in 2020. The baseline stroke risk was high; 2049 patients (7.7%) in 2010 and 1799 patients (8.3%) in 2020 had prior stroke or transient ischemic attack, and the respective mean (SD) CHA_2_DS_2_-VASc scores were 4.4 (1.5) and 4.5 (1.5). The prevalence of risk factors for bleeding was examined; the mean (SD) HAS-BLED score was 3.7 (0.9) to 3.8 (1.0), 14.5% (3877 patients) to 17.4% (8921 patients) were frail, 24.7% (6614 patients) to 28.1% (14 384 patients) had anemia, 2.2% (1027 patients) to 2.8% (922 patients) had a history of hip or pelvic fracture, and 8.0% (2311 patients) to 9.7% (2596 patients) had osteoporosis.

**Table 1.** Characteristics of Oral Anticoagulant–Eligible Incident Atrial Fibrillation Cohort, 2010-2020

Characteristics	Patients, No. (%)										
	2010 (n = 26 782)	2011 (n = 28 275)	2012 (n = 28 136)	2013 (n = 28 544)	2014 (n = 28 755)	2015 (n = 33 443)	2016 (n = 37 659)	2017 (n = 46 018)	2018 (n = 51 236)	2019 (n = 51 037)	2020 (n = 21 603)
Age, mean (SD), y	77.4 (6.0)	77.2 (6.1)	77.2 (6.3)	77.3 (6.4)	77.3 (6.4)	77.3 (6.5)	77.4 (6.6)	77.3 (6.6)	77.4 (6.8)	77.3 (6.9)	77.2 (7.0)
65-79	17 551 (65.5)	17 087 (60.4)	15 713 (55.8)	15 808 (55.4)	16 260 (56.5)	19 138 (57.2)	21 809 (57.9)	27 799 (60.4)	30 530 (59.6)	31 131 (61.0)	13 346 (61.8)
≥80	9231 (34.5)	11 188 (39.6)	12 423 (44.2)	12 736 (44.6)	12 495 (43.5)	14 305 (42.8)	15 850 (42.1)	18 219 (39.6)	20 706 (40.4)	19 906 (39.0)	8257 (38.2)
Sex											
Female	13 871 (51.8)	14 572 (51.5)	14 324 (50.9)	14 574 (51.1)	14 438 (50.2)	17 011 (50.9)	18 876 (50.1)	22 901 (49.8)	25 837 (50.4)	25 725 (50.4)	10 770 (49.9)
Male	12 911 (48.2)	13 703 (48.5)	13 812 (49.1)	13 970 (48.9)	14 317 (49.8)	16 432 (49.1)	18 783 (49.9)	23 117 (50.2)	25 399 (49.6)	25 312 (49.6)	10 833 (50.1)
Race and ethnicity											
Asian	558 (2.1)	595 (2.1)	575 (2.0)	810 (2.8)	856 (3.0)	853 (2.6)	949 (2.5)	1103 (2.4)	1146 (2.2)	1146 (2.2)	459 (2.1)
Black	1986 (7.4)	2112 (7.5)	2148 (7.6)	2096 (7.3)	2075 (7.2)	2421 (7.2)	2960 (7.9)	4279 (9.3)	5101 (10.0)	4750 (9.3)	1908 (8.8)
Hispanic	2022 (7.5)	2063 (7.3)	2127 (7.6)	2430 (8.5)	2425 (8.4)	2858 (8.5)	3010 (8.0)	4323 (9.4)	4409 (8.6)	4406 (8.6)	1900 (8.8)
White	21 004 (78.4)	22 133 (78.3)	2 1947 (78.0)	21 968 (77.0)	21 888 (76.1)	25 628 (76.6)	28 849 (76.6)	34 322 (74.6)	38 465 (75.1)	38 366 (75.2)	16 131 (74.7)
Other^a^	1212 (4.5)	1372 (4.9)	1339 (4.8)	1240 (4.3)	1511 (5.3)	1683 (5.0)	1891 (5.0)	1991 (4.3)	2115 (4.1)	2369 (4.6)	1205 (5.6)
Combined Comorbidity Index, mean (SD)	2.7 (2.3)	2.8 (2.4)	2.8 (2.4)	2.8 (2.5)	2.9 (2.5)	3.0 (2.5)	3.3 (2.8)	3.5 (2.8)	3.6 (2.9)	3.6 (2.9)	3.7 (2.9)
CHA_2_DS_2_-VASc score, mean (SD)	4.4 (1.5)	4.4 (1.5)	4.4 (1.5)	4.5 (1.5)	4.4 (1.5)	4.4 (1.5)	4.4 (1.5)	4.5 (1.5)	4.5 (1.5)	4.5 (1.5)	4.5 (1.5)
HAS-BLED score, mean (SD)^b^	3.7 (0.9)	3.7 (0.9)	3.7 (0.9)	3.7 (0.9)	3.7 (0.9)	3.7 (0.9)	3.7 (0.9)	3.7 (0.9)	3.8 (0.9)	3.8 (0.9)	3.8 (1.0)
Cardiovascular comorbidities											
Chronic kidney disease	5284 (19.7)	6303 (22.3)	6608 (23.5)	6945 (24.3)	7275 (25.3)	9105 (27.2)	11 710 (31.1)	15 429 (33.5)	17 829 (34.8)	18 366 (36.0)	7905 (36.6)
Diabetes	7784 (29.1)	8651 (30.6)	8850 (31.5)	9269 (32.5)	9369 (32.6)	10 998 (32.9)	12 317 (32.7)	15 808 (34.4)	17 849 (34.8)	17 857 (35.0)	7407 (34.3)
Heart failure	6094 (22.8)	6396 (22.6)	6194 (22.0)	6333 (22.2)	6385 (22.2)	7492 (22.4)	7873 (20.9)	10 142 (22.0)	11 577 (22.6)	11 561 (22.7)	5163 (23.9)
Hypertension	21 650 (80.8)	23 195 (82.0)	23 307 (82.8)	23 364 (81.9)	23 450 (81.6)	27 521 (82.3)	31 475 (83.6)	38 453 (83.6)	43 780 (85.4)	43 685 (85.6)	18 310 (84.8)
Ischemic heart disease	10 890 (40.7)	11 297 (40.0)	11 090 (39.4)	11 207 (39.3)	11 030 (38.4)	12 583 (37.6)	14 473 (38.4)	17 738 (38.5)	20 269 (39.6)	20 193 (39.6)	8606 (39.8)
Peripheral vascular disease	4800 (17.9)	5172 (18.3)	5045 (17.9)	5220 (18.3)	5477 (19.0)	6752 (20.2)	9633 (25.6)	12 504 (27.2)	14 535 (28.4)	14 563 (28.5)	6250 (28.9)
Stroke or transient ischemic attack	2049 (7.7)	2078 (7.3)	2067 (7.3)	2160 (7.6)	2287 (8.0)	2755 (8.2)	3058 (8.1)	3808 (8.3)	4365 (8.5)	4173 (8.2)	1799 (8.3)
Noncardiovascular comorbidities											
Alzheimer disease or dementia	2782 (10.4)	2952 (10.4)	2780 (9.9)	2926 (10.3)	2915 (10.1)	3322 (9.9)	4127 (11.0)	5181 (11.3)	6053 (11.8)	5774(11.3)	2514 (11.6)
Anemia	6614 (24.7)	7165 (25.3)	7323 (26.0)	7517 (26.3)	7437 (25.9)	8558 (25.6)	9735 (25.9)	12 078 (26.2)	14 384 (28.1)	14 144 (27.7)	5970 (27.6)
Asthma	1701 (6.4)	1774 (6.3)	1760 (6.3)	1908 (6.7)	1904 (6.6)	2380 (7.1)	2439 (6.5)	2934 (6.4)	3484 (6.8)	3665 (7.2)	1458 (6.7)
Chronic obstructive pulmonary disease	4990 (18.6)	5556 (19.6)	5366 (19.1)	5405 (18.9)	5483 (19.1)	6439 (19.3)	7393 (19.6)	9231 (20.1)	10 454 (20.4)	10 472 (20.5)	4320 (20.0)
Cancer	3159 (11.8)	3265 (11.5)	3251 (11.6)	3231 (11.3)	3107 (10.8)	3816 (11.4)	4529 (12.0)	5454 (11.9)	6524 (12.7)	6579 (12.9)	2659 (12.3)
Depression	2983 (11.1)	3339 (11.8)	3414 (12.1)	3532 (12.4)	3911 (13.6)	4883 (14.6)	6011 (16.0)	7815 (17.0)	9326 (18.2)	9791 (19.2)	4106 (19.0)
Frailty	3877 (14.5)	4288 (15.2)	4151 (14.8)	4297 (15.1)	4305 (15.0)	5033 (15.0)	5872 (15.6)	7393 (16.1)	8921 (17.4)	8619 (16.9)	3585 (16.6)
Hip or pelvic fracture	730 (2.7)	722 (2.6)	744 (2.6)	797 (2.8)	798 (2.8)	922 (2.8)	929 (2.5)	1027 (2.2)	1227 (2.4)	1219 (2.4)	494 (2.3)
Osteoporosis	2596 (9.7)	2647 (9.4)	2492 (8.9)	2381 (8.3)	2311 (8.0)	2730 (8.2)	3109 (8.3)	3779 (8.2)	4468 (8.7)	4683 (9.2)	2000 (9.3)
Rheumatoid or osteoarthritis	8979 (33.5)	9687 (34.3)	9595 (34.1)	9497 (33.3)	9618 (33.4)	11 801 (35.3)	14 195 (37.7)	18 227 (39.6)	21 363 (41.7)	21 302 (41.7)	8915 (41.3)

Abbreviations: CHA_2_DS_2_-VASc, 1 point for congestive heart failure, 1 point for hypertension, 2 points for age 75 years or older, 1 point for age 65 to 74 years, 1 point for diabetes, 2 points for history of stroke or transient ischemic attack or systemic thromboembolism, 1 point for vascular disease including myocardial infarction or peripheral arterial disease, and 1 point for female sex; HAS-BLED, 1 point each for hypertension, renal disease, liver disease, prior stroke, prior history of bleeding, age greater than 65, use of aspirin and other antiplatelets, and alcohol use disorder.

^a^ Includes American Indian, Pacific Islander, any other race, and unknown.

^b^ Excluded labile international normalized ratio component.

### Trends in OAC Initiation and DOAC Uptake

**Figure 2.**
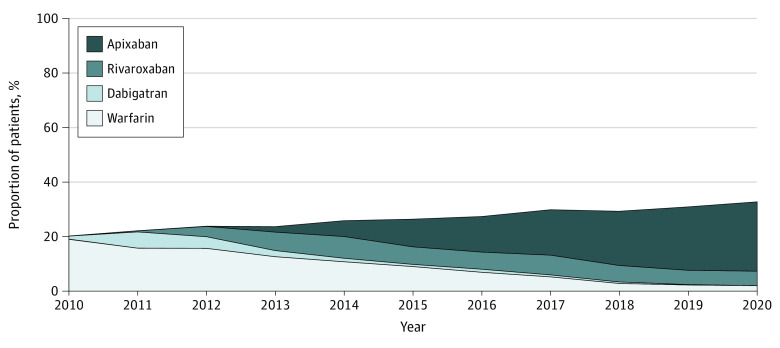
Oral Anticoagulant (OAC) Initiation and Direct OAC Uptake in 2010-2020 The stacked area plots depict the proportion of 12-month OAC initiation and direct OAC uptake in the total OAC-eligible incident atrial fibrillation cohort. OAC initiation increased from 20.2% to 32.9% (odds ratio for OAC use per year, 1.06; 95% CI, 1.06-1.07; *P* < .001).

**Figure 3.**
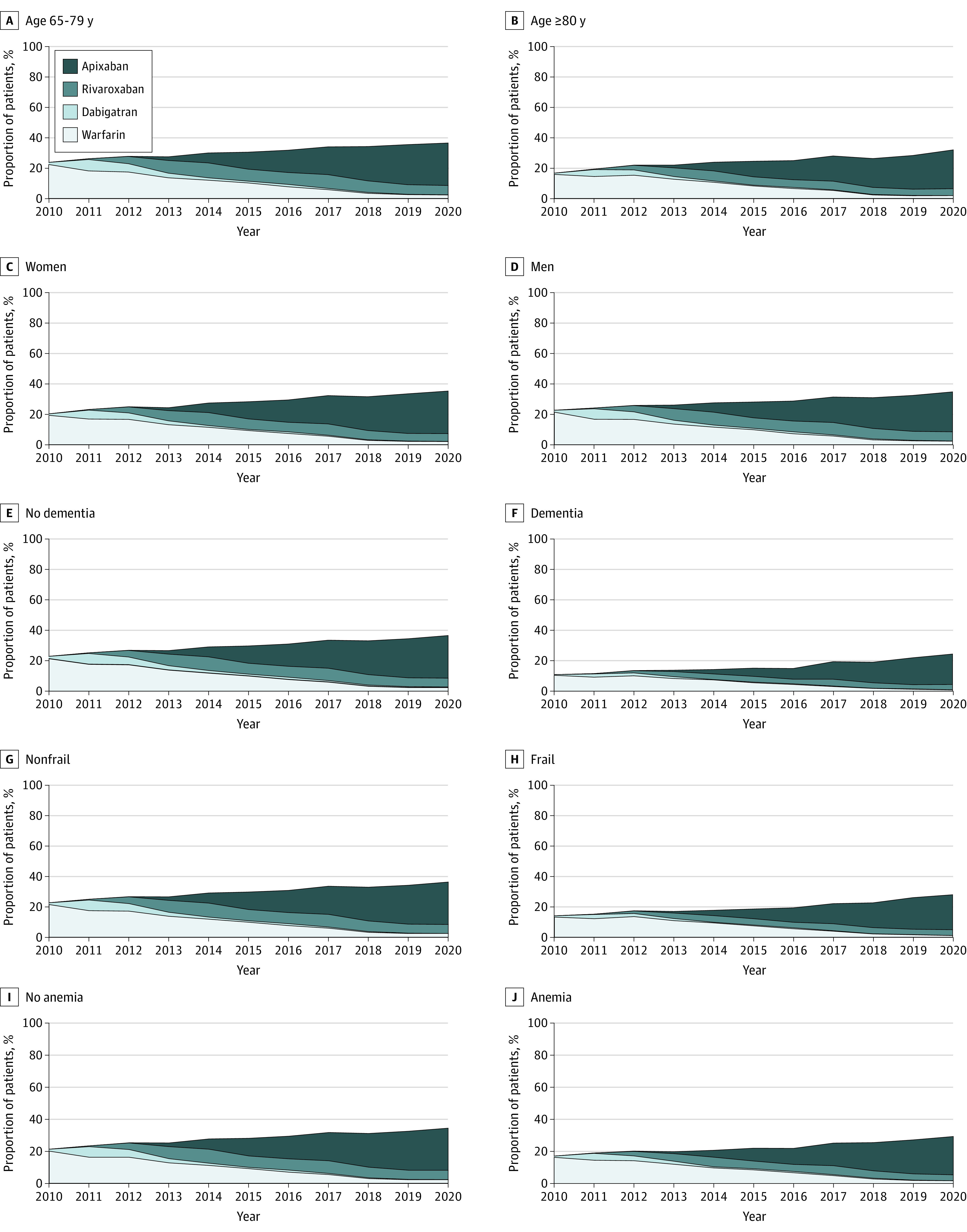
Oral Anticoagulant (OAC) Initiation and Direct OAC Uptake by Subgroups in 2010-2020 The stacked area plots depict the proportion of 12-month OAC initiation and direct OAC uptake in the OAC-eligible incident AF cohort stratified by age (A and B), sex (C and D), dementia (E and F), frailty (G and H), and anemia (I and J). There was an increase in OAC initiation in patients aged 65 to 79 years and 80 years and older, in women and men, in patients with dementia and without dementia, in frail and nonfrail patients, and in patients with anemia and without anemia (*P* < .001 for all).

Among patients with new diagnoses of AF (CHA_2_DS_2_-VASc score ≥2 for men, CHA_2_DS_2_-VASc score ≥3 for women) and eligible for OAC, there was a 12.7% increase in the proportion of patients who were newly prescribed OAC within 1 year of the AF diagnosis, from 20.2% (5405 of 26 782 patients) in 2010 to 32.9% (7111 of 21 603 patients) in 2020 (odds ratio [OR] for OAC initiation per year, 1.06; 95% CI, 1.06-1.07; *P* < .001) (Figure 2 and eTable 1 in the Supplement). DOAC uptake increased from 1.1% (291 of 26 782 patients) to 30.9% (6678 of 21 603 patients), and warfarin initiation decreased from 19.1% (5114 of 26 782 patients) to 2.0% (436 of 21 603 patients). In 2020, 67.1% of MA plan beneficiaries did not receive any OAC within 12 months of the AF diagnosis. Among patients who were initiated on OAC, there was an increase in DOAC uptake from 5.4% (291 of 5405 patients) in 2010 to 93.9% (6678 of 7111 patients) in 2020 (OR for initiation of DOAC vs warfarin per year, 1.62; 95% CI, 95% CI, 1.61-1.63; *P* < .001). Since 2017, factor Xa inhibitors (rivaroxaban and apixaban) have been the dominant class of OAC. Among patients with a new AF diagnosis in 2020 who were prescribed OAC within 12 months, apixaban was the most commonly prescribed OAC (5528 patients [77.7%]), followed by rivaroxaban (1135 patients [16.0%]), warfarin (436 patients [6.1%]), and dabigatran (15 patients [0.2%]). Throughout the study period, OAC initiation was lower in patients aged 80 years and older and in patients with dementia, frailty, and anemia (Figure 3 and eTable 1 in the Supplement). The OAC initiation and DOAC uptake were similar across subgroups by race and ethnicity, HAS-BLED, and prior stroke (eFigure in the Supplement).

### Patient Characteristics Associated With Noninitiation of OAC and DOACs

Characteristics of the OAC-eligible incident AF cohort stratified by OAC status are shown in Table 2. Of 381 488 patients with newly diagnosed AF and with CHA_2_DS_2_-VASc score 2 or higher for men and 3 or higher for women, 27.1% (103 537 patients) were prescribed OAC within 12 months of the AF diagnosis. In the multivariable analyses comparing OAC users with nonusers (Table 2), older age (OR, 0.98; 95% CI, 0.98-0.98), dementia (OR, 0.57; 95% CI, 0.55-0.58), frailty (OR, 0.74; 95% CI, 0.72-0.76), anemia (OR, 0.75; 95% CI, 0.74-0.77), and history of hip or pelvic fracture (OR, 0.90; 95% CI, 0.85-0.94) were associated with lower odds of OAC initiation. Greater CHA_2_DS_2_-VASc score and high HAS-BLED score were both associated with greater odds of OAC initiation. Among patients who were initiated on OAC, female sex and higher HAS-BLED score were associated with greater odds of DOAC initiation. Anemia, prior hip or pelvic fracture, and chronic kidney disease were associated with lower odds of DOAC initiation.

**Table 2.** Characteristics of OAC-Eligible Incident Atrial Fibrillation Cohort and Their Association With Initiation of OACs and DOAC, 2010-2020

Characteristics	Warfarin (n = 31 638)	DOAC (n = 71 899)^a^	None (n = 277 951)	OAC vs none, OR (95% CI)	DOAC vs warfarin, OR (95% CI)
DOAC					
Dabigatran	NA	5642 (7.8)	NA	NA	NA
Rivaroxaban	NA	20 230 (28.1)	NA	NA	NA
Apixaban	NA	46 035 (64.0)	NA	NA	NA
Age, mean (SD)	76.7 (6.0)	76.5 (6.4)	77.6 (6.7)	0.98 (0.98-0.98)	0.99 (0.99-1.00)
Sex					
Female	15 697 (49.6)	36 402 (50.6)	140 800 (50.7)	1.02 (1.00-1.04)	1.06 (1.02-1.11)
Male	15 941 (50.4)	35 497 (49.4)	137 151 (49.3)	1 [Reference]	1 [Reference]
Race					
Asian	667 (2.1)	1729 (2.4)	6654 (2.4)	0.95 (0.91-1.00)	1.13 (1.02-1.26)
Black	2228 (7.0)	6105 (8.5)	23 503 (8.5)	0.90 (0.88-0.93)	1.15 (1.08-1.22)
Hispanic	2780 (8.8)	6387 (8.9)	22 806 (8.2)	1.06 (1.04-1.09)	0.96 (0.90-1.01)
White	24 495 (77.4)	54 523 (75.8)	211 683 (76.2)	1 [Reference]	1 [Reference]
Other^b^	1468 (4.6)	3155 (4.4)	13 305 (4.8)	0.93 (0.89-0.96)	0.93 (0.87-1.01)
Combined Comorbidity Index, mean (SD)	3.0 (2.5)	3.1 (2.6)	3.3 (2.7)	1.00 (0.99-1.00)	0.97 (0.95-0.98)
CHA_2_DS_2_-VASc score, mean (SD)	4.5 (1.5)	4.4 (1.5)	4.4 (1.5)	1.03 (1.02-1.04)	0.96 (0.94-0.98)
HAS-BLED score ≥4^c^	17 461 (55.2)	40 630 (56.5)	150 705 (54.2)	1.17 (1.15-1.19)	1.12 (1.08-1.17)
Cardiovascular comorbidities					
Chronic kidney disease	8950 (28.3)	21 938 (30.5)	81 871 (29.5)	1.05 (1.03-1.08)	0.83 (0.80-0.87)
Diabetes	10 892 (34.4)	24 137 (33.6)	91 130 (32.8)	0.99 (0.97-1.01)	0.98 (0.94-1.02)
Heart failure	8647 (27.3)	16 366 (22.8)	60 197 (21.7)	1.29 (1.26-1.32)	0.84 (0.80-0.89)
Hypertension	26 260 (83.0)	61 723 (85.8)	230 207 (82.8)	1.12 (1.10-1.15)	1.31 (1.25-1.37)
Ischemic heart disease	12 143 (38.4)	25 805 (35.9)	111 428 (40.1)	0.82 (0.80-0.83)	0.97 (0.93-1.01)
Peripheral vascular disease	6336 (20.0)	16 646 (23.2)	66 969 (24.1)	0.90 (0.88-0.91)	1.05 (1.00-1.09)
Stroke or transient ischemic attack	3172 (10.0)	6278 (8.7)	21 149 (7.6)	1.24 (1.21-1.28)	0.87 (0.82-0.93)
Noncardiovascular comorbidities					
Alzheimer disease or dementia	1804 (5.7)	4438 (6.2)	35 084 (12.6)	0.57 (0.55-0.58)	1.07 (0.99-1.16)
Anemia	7532 (23.8)	15 795 (22.0)	77 598 (27.9)	0.75 (0.74-0.77)	0.86 (0.82-0.89)
Asthma or chronic obstructive pulmonary disease	2134 (6.7)	5012 (7.0)	18 261 (6.6)	1.00 (0.98-1.02)	0.97 (0.93-1.01)
Cancer	3501 (11.1)	8542 (11.9)	33 531 (12.1)	0.98 (0.96-1.00)	1.10 (1.04-1.16)
Depression	3865 (12.2)	10 511 (14.6)	44 735 (16.1)	0.91 (0.89-0.93)	0.95 (0.90-1.00)
Frailty	3587 (11.3)	8013 (11.1)	48 741 (17.5)	0.74 (0.72-0.76)	1.00 (0.94-1.06)
Hip or pelvic fracture	696 (2.2)	1196 (1.7)	7717 (2.8)	0.90 (0.85-0.94)	0.78 (0.70-0.88)
Osteoporosis	2466 (7.8)	5859 (8.1)	24 871 (8.9)	0.99 (0.96-1.01)	1.08 (1.02-1.15)
Rheumatoid or ostearthritis	10 999 (34.8)	27 927 (38.8)	104 253 (37.5)	1.03 (1.02-1.05)	1.06 (1.02-1.09)

Abbreviations: CHA_2_DS_2_-VASc, 1 point for congestive heart failure, 1 point for hypertension, 2 points for age 75 years or older, 1 point for age 65 to 74 years, 1 point for diabetes, 2 points for history of stroke or transient ischemic attack or systemic thromboembolism, 1 point for vascular disease including myocardial infarction or peripheral arterial disease, and 1 point for female sex; DOAC, direct oral anticoagulant; HAS-BLED, 1 point each for hypertension, renal disease, liver disease, prior stroke, prior history of bleeding, age greater than 65, use of aspirin and other antiplatelets, and alcohol use disorder; NA, not applicable; OAC, oral anticoagulant; OR, odds ratio.

^a^ Eight patients were prescribed both rivaroxaban and apixaban on the same day.

^b^ Includes American Indian, Pacific Islander, any other race, and unknown.

^c^ Excluded labile international normalized ratio component.

### Trends in OAC Nonadherence

Over time, the median (IQR) PDC increased from 77.6% (41.0%-96.4%) to 90.2% (57.4%-96.6%), and the prevalence of OAC nonadherence decreased by 13.2%, from 52.2% (2290 of 4389 patients) to 39.0% (3434 of 8798 patients). The same pattern was observed in all the subgroups (eTable 2 in the Supplement). Throughout the study period, the prevalence of OAC nonadherence was consistently higher in patients with dementia, frailty, anemia, and higher HAS-BLED score. Greatest reductions in OAC nonadherence were observed in patients with low CHA_2_DS_2_-VASc score (–17.6%) and aged 80 years or older (–16.0%).

## Discussion

To our knowledge, this cohort study provides the most contemporary national OAC prescription patterns for AF in the US. There are 4 main findings of our study. First, after the introduction of DOACs, the rate of 12-month initiation of OAC among patients with newly diagnosed AF increased by 12.7% from 2010 to 2020. Second, among OAC initiators, apixaban is now the most widely prescribed OAC. Third, initiation of OAC remains suboptimal in older adults with AF, particularly among those with dementia, frailty, and anemia. Fourth, since the introduction of DOACs, adherence with OAC has improved.

Similar to the prior study of Medicare FFS beneficiaries by Norby et al,^12^ in the current study of MA plan beneficiaries, OAC initiation improved significantly in the past decade after the introduction of DOACs. However, in both studies, OAC initiation remained suboptimal, with 47% of Medicare FFS beneficiaries in 2016^12^ and 67.1% of MA plan beneficiaries in 2020 not receiving any OAC at any time after the incident AF diagnosis or within 12 months. We observed that the size of the cohort increased as a result of increased enrollment in MA plans, which has now reached nearly 50% of all Medicare beneficiaries. Additionally, we examined OAC initiation in high-risk patients with dementia, frailty, and anemia, and our findings suggest that the availability of DOACs has improved OAC initiation in all older adults with AF and particularly in patients at high risk of bleeding, but a persistent practice gap remains. Finally, our study shows that in parallel with greater DOAC uptake, OAC adherence has improved in the past decade. This result is consistent with a prior study^26^ showing greater adherence with DOAC than with warfarin and patients initiated on apixaban being most likely to adhere to OAC.

The availability of DOACs has also improved OAC initiation in other countries. In Denmark, 6-month initiation of OAC after incident AF among adults aged 30 years or older increased from less than 40% in 2005 to 66.5% in 2015.^27^ In Alberta, Canada, 90-day initiation of OAC after incident AF in adults aged 18 years or older with CHA_2_DS_2_-VASc score greater than 2 in men and greater than 3 in women increased from 44% in 2008 to 67% in 2019.^28^ The rate of OAC initiation in our study was comparatively lower, which likely reflects differences in health care utilization across widely different health care systems.

The association between noninitiation of OAC in older adults with concomitant geriatric conditions highlights the frequent challenges faced by clinicians in optimizing the risks and benefits of OAC in a medically complex older population. The fear of adverse events associated with OAC has been cited as the most important reason for lack of OAC prescription in patients with advanced age and high comorbidity burden.^29^ In a recent survey by the Heart Rhythm Society AF Centers of Excellence Task Force,^30^ patient’s prior bleeding and risk of bleeding were the most important barriers to initiation of OAC. DOACs do not eliminate bleeding risk, and in the pivotal trials, the annual risks of major bleeding in adults aged 75 years and older were 3.3% and 4.9% in the apixaban and rivaroxaban groups, respectively.^31,32^ Moreover, the risk of major bleeding was twice as high in patients with 6 or more comorbidities compared with patients with 0 to 2 comorbidities.^33^ For effective and safe AF management, a multidisciplinary, team-based approach including patients and their caregivers, primary care physicians, geriatricians, general cardiologists, electrophysiologists, cardiovascular pharmacists, and other stakeholders has been proposed in the US.^34^ This effort may improve OAC initiation in patients who were previously considered to have high bleeding risk. Left atrial appendage occlusion may be offered for patients with untenable bleeding risk,^35,36^ although the long-term safety and effectiveness of this new invasive procedure compared with OAC for highly frail and complex patients in routine care is unclear. Several factor XIa inhibitors are currently undergoing phase 2 trials,^37,38^ and if proven to reduce bleeding risks compared with factor Xa inhibitors, this new class of anticoagulants may address this large unmet clinical need.

The professional guidelines have recommended all DOACs on an equal footing since their regulatory approval,^23,39^ but apixaban has been clearly preferred by prescribers in both North America and Europe^12,28,40^ for several reasons.^41^ Dabigatran was the first DOAC to be approved for AF, but concerns regarding its bleeding risks limited its uptake, even after the US Food and Drug Administration approval of idaricuzimab in 2015.^42^ Rivaroxaban and dabigatran were both associated with greater risk of gastrointestinal bleeding compared with warfarin in the pivotal trials.^43,44^ Apixaban was the only DOAC that was shown to have a risk of major bleeding that is lower than that of warfarin^45^ and not significantly higher than that of aspirin.^46^ Although apixaban and rivaroxaban have similar half-lives, rivaroxaban as a once-daily drug has greater variability in peak and trough concentration^47^ and may be associated with greater risk of both ischemic and bleeding events.^48^Additionally, compared with rivaroxaban (37% renal clearance) and dabigatran (80% renal clearance), apixaban (27% renal clearance) is less dependent on renal clearance and has more predictable pharmacokinetics in patients with reduced renal function.^49^ A recent analysis of FFS Medicare beneficiaries showed that only apixaban, not dabigatran or rivaroxaban, was associated with lower rates of composite clinical events, in particular, major bleeding, than warfarin across all frailty levels.^50^ Edoxaban uptake was only 0.01% to 0.02% in our study, likely because, as the fourth DOAC to be approved, it entered a saturated market and was initially approved only for patients with AF and renal dysfunction.^42^

### Limitations

The results of our study should be interpreted in the contexts of its limitations. First, as we described previously,^12^ there are differences in the rate of OAC initiation between Medicare FFS and MA plan beneficiaries, and, therefore, our data may not be generalizable to older adults who are not enrolled in MA plans. Nevertheless, given that the vast majority of OAC-eligible AF patients are aged 65 years or older and 42% of Medicare beneficiaries were enrolled in MA plans in 2020,^13^ our study adds important data to identify the national practice patterns in OAC prescription. Second, our analysis was restricted to patients who survived and had the insurance coverage for the entire calendar year so that we could assess OAC exposure and adherence for the year. This population is likely to exclude patients who switched their insurance during the year or were near the end of life. Third, our study using administrative claims data most likely underestimated prevalence of important risk factors for bleeding that may affect OAC initiation, such as history of falls and over-the-counter antiplatelet or nonsteroidal anti-inflammatory drug use. Fourth, our analysis includes data up to 2020, and the OAC initiation and DOAC uptake may have increased further after the 2019 endorsement of DOACs as safer alternatives to warfarin by the US guideline.^39^ Fifth, the transition from *International Classification of Diseases, Ninth Revision *to *International Statistical Classification of Diseases and Related Health Problems, Tenth Revision *in October 2015 may have affected the analysis of temporal trends. Sixth, our assumptions of OAC underutilization do not take into account patient preferences.

## Conclusions

Our cohort study demonstrates that since the introduction of DOACs, OAC initiation within 12 months of new AF diagnosis has improved in older adults. Nevertheless, substantial practice gap remains, with 67.1% of patients with incident AF in 2020 not being started on OAC within 12 months of the AF diagnosis. Patients with dementia, frailty, and anemia were persistently undertreated with OAC. Additional strategies are needed to improve OAC utilization in patients at high bleeding and stroke risks and to develop alternative strategies for stroke prophylaxis in patients with untenable bleeding risk.

## Notes

Supplement. **eTable 1.** 12-Month Initiation of Oral Anticoagulant in Oral Anticoagulant-Eligible Incident Atrial Fibrillation Cohort, 2010-2020 **eTable 2.** OAC Mean Proportion of Days Covered and Nonadherence (Proportion of Days Covered < 80%) in OAC-Initiator Incident Atrial Fibrillation Cohort, 2010-2020 **eFigure.** OAC Initiation and DOAC Uptake by Race and Additional Clinical Subgroups Over 2010-2020
