# Supplementary material for: Trends in Use of Oral Anticoagulants in Older Adults With Newly Diagnosed Atrial Fibrillation, 2010-2020
Source: JAMA Netw Open. 2022 Nov 18;5(11):e2242964. doi: 10.1001/jamanetworkopen.2022.42964 (PMC9675002; doi:10.1001/jamanetworkopen.2022.42964)
Supplement: Supplement. — eTable 1. 12-Month Initiation of Oral Anticoagulant in Oral Anticoagulant-Eligible Incident Atrial Fibrillation Cohort, 2010-2020 eTable 2. OAC Mean Proportion of Days Covered and Nonadherence (Proportion of Days Covered < 80%) in OAC-Initiator Incident Atrial Fibrillation Cohort, 2010-2020 eFigure. OAC Initiation and DOAC Uptake by Race and Additional Clinical Subgroups Over 2010-2020 [file jamanetwopen-e2242964-s001.pdf]

Supplemental Online Content

Ko D, Lin KJ, Bessette LG, et al. Trends in use of oral anticoagulants in older adults with newly diagnosed atrial fibrillation, 2010-2020. *JAMA Netw Open*. 2022;5(11):e2242964. doi:10.1001/jamanetworkopen.2022.42964

eTable 1. 12-Month Initiation of Oral Anticoagulant in Oral Anticoagulant-Eligible Incident Atrial Fibrillation Cohort, 2010-2020

eTable 2. OAC Mean Proportion of Days Covered and Nonadherence (Proportion of Days Covered < 80%) in OAC-Initiator Incident Atrial Fibrillation Cohort, 2010-2020

eFigure. OAC Initiation and DOAC Uptake by Race and Additional Clinical Subgroups Over 2010-2020

This supplemental material has been provided by the authors to give readers additional information about their work.

© 2022 Ko D et al. *JAMA Network Open.*

eTable 1. 12-Month Initiation of Oral Anticoagulant in Oral Anticoagulant-Eligible Incident Atrial Fibrillation Cohort, 2010-2020

**Characteristics**	**2010**	**2011**	**2012**	**2013**	**2014**	**2015**	**2016**	**2017**	**2018**	**2019**	**2020**
All, N	26782	28275	28136	28544	28755	33443	37659	46018	51236	51037	21603
Dabigatran, n (%)	291 (1.1)	1701 (6.0)	1222 (4.3)	674 (2.4)	349 (1.2)	281 (0.8)	429 (1.1)	313 (0.7)	251 (0.5)	116 (0.2)	15 (0.1)
Rivaroxaban, n (%)	0 (0.0)	118 (0.4)	1066 (3.8)	1932 (6.8)	2300 (8.0)	2176 (6.5)	2377 (6.3)	3367 (7.3)	3111 (6.1)	2648 (5.2)	1135 (5.3)
Apixaban, n (%)	0 (0.0)	0 (0.0)	29 (0.1)	575 (2.0)	1682 (5.8)	3409 (10.2)	4924 (13.1)	7670 (16.7)	10263 (20.0)	11955 (23.4)	5528 (25.6)
Warfarin, n (%)	5114 (19.1)	4463 (15.8)	4402 (15.6)	3577 (12.5)	3104 (10.8)	2984 (8.9)	2579 (6.8)	2432 (5.3)	1449 (2.8)	1098 (2.2)	436 (2.0)
None, %	21377 (79.8)	21993 (77.8)	21417 (76.1)	21786 (76.3)	21320 (74.1)	24594 (73.5)	27350 (72.6)	32237 (70.1)	36164 (70.6)	35221 (69.0)	14492 (67.1)
Age 65-79 years, N	17551	17087	15713	15808	16260	19138	21809	27799	30530	31131	13346
Dabigatran, n (%)	221 (1.3)	1218 (7.1)	806 (5.1)	451 (2.9)	247 (1.5)	203 (1.1)	303 (1.4)	234 (0.8)	180 (0.6)	86 (0.3)	11 (0.1)
Rivaroxaban, n (%)	0 (0.0)	83 (0.5)	711 (4.5)	1249 (7.9)	1510 (9.3)	1438 (7.5)	1624 (7.4)	2381 (8.6)	2196 (7.2)	1863 (6.0)	777 (5.8)
Apixaban, n (%)	0 (0.0)	0 (0.0)	18 (0.1)	364 (2.3)	1008 (6.2)	2026 (10.6)	3042 (13.9)	4815 (17.3)	6542 (21.4)	7777 (25.0)	3533 (26.5)
Warfarin, n (%)	3729 (21.2)	2929 (17.1)	2605 (16.6)	2051 (13.0)	1848 (11.4)	1864 (9.7)	1600 (7.3)	1525 (5.5)	989 (3.2)	750 (2.4)	293 (2.2)
None, n (%)	13601 (77.5)	12857 (75.2)	11573 (73.7)	11693 (74.0)	11647 (71.6)	13608 (71.1)	15240 (69.9)	18845 (67.8)	20624 (67.6)	20656 (66.4)	8733 (65.4)
Age ≥80 years, N	9231	11188	12423	12736	12495	14305	15850	18219	20706	19906	8257
Dabigatran, n (%)	70 (0.8)	483 (4.3)	416 (3.3)	223 (1.8)	102 (0.8)	78 (0.5)	126 (0.8)	79 (0.4)	71 (0.3)	30 (0.2)	4 (0.0)
Rivaroxaban, n (%)	0 (0.0)	35 (0.3)	355 (2.9)	683 (5.4)	790 (6.3)	738 (5.2)	753 (4.7)	986 (5.4)	915 (4.4)	785 (3.9)	358 (4.3)
Apixaban, n (%)	0 (0.0)	0 (0.0)	11 (0.1)	211 (1.7)	674 (5.4)	1383 (9.7)	1882 (11.9)	2855 (15.7)	3721 (18.0)	4178 (21.0)	1995 (24.2)
Warfarin, n (%)	1385 (15.0)	1534 (13.7)	1797 (14.5)	1526 (12.0)	1256 (10.1)	1120 (7.8)	979 (6.2)	907 (5.0)	460 (2.2)	348 (1.7)	143 (1.7)
None, n (%)	7776 (84.2)	9136 (81.7)	9844 (79.2)	10093 (79.2)	9673 (77.4)	10986 (76.8)	12110 (76.4)	13392 (73.5)	15540 (75.1)	14565 (73.2)	5759 (69.7)
Women, N	13871	14572	14324	14574	14438	17011	18876	22901	25837	25725	10770
Dabigatran, n (%)	131 (0.9)	812 (5.6)	571 (4.0)	338 (2.3)	165 (1.1)	127 (0.7)	202 (1.1)	142 (0.6)	111 (0.4)	49 (0.2)	7 (0.1)
Rivaroxaban, n (%)	0 (0.0)	52 (0.4)	526 (3.7)	922 (6.3)	1149 (8.0)	1106 (6.5)	1113 (5.9)	1597 (7.0)	1469 (5.7)	1218 (4.7)	516 (4.8)
Apixaban, n (%)	0 (0.0)	0 (0.0)	14 (0.1)	267 (1.8)	848 (5.9)	1803 (10.6)	2601 (13.8)	4006 (17.5)	5401 (20.9)	6308 (24.5)	2836 (26.3)
Warfarin, n (%)	2505 (18.1)	2294 (15.7)	2234 (15.6)	1795 (12.3)	1555 (10.8)	1464 (8.6)	1300 (6.9)	1189 (5.2)	667 (2.6)	490 (1.9)	204 (1.9)
None, n (%)	11235 (81.0)	11414 (78.3)	10979 (76.6)	11252 (77.2)	10721 (74.3)	12511 (73.5)	13660 (72.4)	15968 (69.7)	18190 (70.4)	17660 (68.6)	7210 (66.9)
Men, N	12911	13703	13812	13970	14317	16432	18783	23117	25399	25312	10833
Dabigatran, n (%)	160 (1.2)	889 (6.5)	651 (4.7)	336 (2.4)	184 (1.3)	154 (0.9)	227 (1.2)	171 (0.7)	140 (0.6)	67 (0.3)	8 (0.1)
Rivaroxaban, n (%)	0 (0.0)	66 (0.5)	540 (3.9)	1010 (7.2)	1151 (8.0)	1070 (6.5)	1264 (6.7)	1770 (7.7)	1642 (6.5)	1430 (5.6)	619 (5.7)
Apixaban, n (%)	0 (0.0)	0 (0.0)	15 (0.1)	308 (2.2)	834 (5.8)	1606 (9.8)	2323 (12.4)	3664 (15.8)	4862 (19.1)	5647 (22.3)	2692 (24.8)
Warfarin, n (%)	2609 (20.2)	2169 (15.8)	2168 (15.7)	1782 (12.8)	1549 (10.8)	1520 (9.3)	1279 (6.8)	1243 (5.4)	782 (3.1)	608 (2.4)	232 (2.1)
None, n (%)	10142 (78.6)	10579 (77.2)	10438 (75.6)	10534 (75.4)	10599 (74.0)	12083 (73.5)	13690 (72.9)	16269 (70.4)	17974 (70.8)	17561 (69.4)	7282 (67.2)

© 2022 Ko D et al. *JAMA Network Open.*

**Characteristics**	**2010**	**2011**	**2012**	**2013**	**2014**	**2015**	**2016**	**2017**	**2018**	**2019**	**2020**
Asian, N	558	595	575	810	856	853	949	1103	1146	1146	459
Dabigatran, n (%)	6 (1.1)	32 (5.4)	18 (3.1)	31 (3.8)	17 (2.0)	12 (1.4)	16 (1.7)	12 (1.1)	5 (0.4)	3 (0.3)	0 (0.0)
Rivaroxaban, n (%)	0 (0.0)	0 (0.0)	25 (4.3)	57 (7.0)	60 (7.0)	62 (7.3)	65 (6.8)	102 (9.2)	95 (8.3)	56 (4.9)	20 (4.4)
Apixaban, n (%)	0 (0.0)	0 (0.0)	0 (0.0)	14 (1.7)	45 (5.3)	74 (8.7)	131 (13.8)	163 (14.8)	236 (20.6)	252 (22.0)	120 (26.1)
Warfarin, n (%)	102 (18.3)	77 (12.9)	86 (15.0)	88 (10.9)	70 (8.2)	67 (7.9)	54 (5.7)	58 (5.3)	25 (2.2)	30 (2.6)	10 (2.2)
None, n (%)	450 (80.6)	486 (81.7)	446 (77.6)	620 (76.5)	664 (77.6)	638 (74.8)	683 (72.0)	768 (69.6)	785 (68.5)	805 (70.2)	309 (67.3)
Black, N	1986	2112	2148	2096	2075	2421	2960	4279	5101	4750	1908
Dabigatran, n (%)	20 (1.0)	128 (6.1)	90 (4.2)	43 (2.1)	24 (1.2)	13 (0.5)	23 (0.8)	14 (0.3)	17 (0.3)	10 (0.2)	0 (0.0)
Rivaroxaban, n (%)	0 (0.0)	7 (0.3)	84 (3.9)	108 (5.2)	127 (6.1)	130 (5.4)	169 (5.7)	299 (7.0)	308 (6.0)	223 (4.7)	91 (4.8)
Apixaban, n (%)	0 (0.0)	0 (0.0)	1 (0.0)	36 (1.7)	120 (5.8)	239 (9.9)	394 (13.3)	745 (17.4)	1075 (21.1)	1100 (23.2)	468 (24.5)
Warfarin, n (%)	361 (18.2)	352 (16.7)	340 (15.8)	263 (12.5)	174 (8.4)	193 (8.0)	186 (6.3)	181 (4.2)	87 (1.7)	63 (1.3)	28 (1.5)
None, n (%)	1605 (80.8)	1625 (76.9)	1633 (76.0)	1646 (78.5)	1630 (78.6)	1846 (76.2)	2188 (73.9)	3040 (71.0)	3615 (70.9)	3354 (70.6)	1321 (69.2)
Hispanic, N	2022	2063	2127	2430	2425	2858	3010	4323	4409	4406	1900
Dabigatran, n (%)	15 (0.7)	109 (5.3)	79 (3.7)	53 (2.2)	28 (1.2)	24 (0.8)	38 (1.3)	41 (0.9)	25 (0.6)	12 (0.3)	1 (0.1)
Rivaroxaban, n (%)	0 (0.0)	3 (0.1)	75 (3.5)	131 (5.4)	185 (7.6)	202 (7.1)	212 (7.0)	349 (8.1)	323 (7.3)	296 (6.7)	110 (5.8)
Apixaban, n (%)	0 (0.0)	0 (0.0)	3 (0.1)	51 (2.1)	135 (5.6)	273 (9.6)	357 (11.9)	706 (16.3)	944 (21.4)	1102 (25.0)	505 (26.6)
Warfarin, n (%)	408 (20.2)	366 (17.7)	365 (17.2)	363 (14.9)	301 (12.4)	304 (10.6)	215 (7.1)	232 (5.4)	118 (2.7)	76 (1.7)	32 (1.7)
None, n (%)	1599 (79.1)	1585 (76.8)	1605 (75.5)	1832 (75.4)	1776 (73.2)	2055 (71.9)	2188 (72.7)	2995 (69.3)	2999 (68.0)	2920 (66.3)	1252 (65.9)
White, N	21004	22133	21947	21968	21888	25628	28849	34322	38465	38366	16131
Dabigatran, n (%)	237 (1.1)	1,358 (6.1)	975 (4.4)	518 (2.4)	254 (1.2)	221 (0.9)	333 (1.2)	231 (0.7)	191 (0.5)	84 (0.2)	11 (0.1)
Rivaroxaban, n (%)	0 (0.0)	97 (0.4)	827 (3.8)	1555 (7.1)	1828 (8.4)	1691 (6.6)	1821 (6.3)	2488 (7.2)	2283 (5.9)	1953 (5.1)	852 (5.3)
Apixaban, n (%)	0 (0.0)	0 (0.0)	23 (0.1)	454 (2.1)	1308 (6.0)	2666 (10.4)	3800 (13.2)	5751 (16.8)	7594 (19.7)	8983 (23.4)	4141 (25.7)
Warfarin, n (%)	4034 (19.2)	3474 (15.7)	3420 (15.6)	2738 (12.5)	2345 (10.7)	2256 (8.8)	1984 (6.9)	1872 (5.5)	1160 (3.0)	875 (2.3)	337 (2.1)
None, n (%)	16733 (79.7)	17204 (77.7)	16702 (76.1)	16703 (76.0)	16153 (73.8)	18795 (73.3)	20911 (72.5)	23980 (69.9)	27237 (70.8)	26472 (69.0)	10793 (66.9)
Other, N	1212	1372	1339	1240	1511	1683	1891	1991	2115	2369	1205
Dabigatran, n (%)	13 (1.1)	74 (5.4)	60 (4.5)	29 (2.3)	26 (1.7)	11 (0.7)	19 (1.0)	15 (0.8)	13 (0.6)	7 (0.3)	3 (0.2)
Rivaroxaban, n (%)	0 (0.0)	11 (0.8)	55 (4.1)	81 (6.5)	100 (6.6)	91 (5.4)	110 (5.8)	129 (6.5)	102 (4.8)	120 (5.1)	62 (5.1)
Apixaban, n (%)	0 (0.0)	0 (0.0)	2 (0.1)	20 (1.6)	74 (4.9)	157 (9.3)	242 (12.8)	305 (15.3)	414 (19.6)	518 (21.9)	294 (24.4)
Warfarin, n (%)	209 (17.2)	194 (14.1)	191 (14.3)	125 (10.1)	214 (14.2)	164 (9.7)	140 (7.4)	89 (4.5)	59 (2.8)	54 (2.3)	29 (2.4)
None, n (%)	990 (81.7)	1093 (79.7)	1031 (77.0)	985 (79.4)	1097 (72.6)	1260 (74.9)	1380 (73.0)	1454 (73.0)	1528 (72.2)	1670 (70.5)	817 (67.8)
No Dementia, N	24000	25323	25356	25618	25840	30121	33532	40837	45183	45263	19089
Dabigatran, n (%)	279 (1.2)	1644 (6.5)	1176 (4.6)	645 (2.5)	344 (1.3)	268 (0.9)	412 (1.2)	295 (0.7)	242 (0.5)	107 (0.2)	13 (0.1)
Rivaroxaban, n (%)	0 (0.0)	112 (0.4)	1031 (4.1)	1843 (7.2)	2204 (8.5)	2062 (6.8)	2266 (6.8)	3161 (7.7)	2919 (6.5)	2504 (5.5)	1057 (5.5)
Apixaban, n (%)	0 (0.0)	0 (0.0)	25 (0.1)	551 (2.2)	1606 (6.2)	3249 (10.8)	4666 (13.9)	7138 (17.5)	9532 (21.1)	11040 (24.4)	5077 (26.6)
Warfarin, n (%)	4857 (20.2)	4221 (16.7)	4150 (16.4)	3359 (13.1)	2911 (11.3)	2822 (9.4)	2417 (7.2)	2291 (5.6)	1351 (3.0)	1037 (2.3)	418 (2.2)

© 2022 Ko D et al. *JAMA Network Open.*

Characteristics	2010	2011	2012	2013	2014	2015	2016	2017	2018	2019	2020
None, n (%)	18864 (78.6)	19346 (76.4)	18974 (74.8)	19220 (75.0)	18775 (72.7)	21721 (72.1)	23771 (70.9)	27953 (68.5)	31140 (68.9)	30576 (67.6)	12527 (65.6)
Dementia, N	2782	2952	2780	2926	2915	3322	4127	5181	6053	5774	2514
Dabigatran, n (%)	12 (0.4)	57 (1.9)	46 (1.7)	29 (1.0)	5 (0.2)	13 (0.4)	17 (0.4)	18 (0.3)	9 (0.1)	9 (0.2)	2 (0.1)
Rivaroxaban, n (%)	0 (0.0)	6 (0.2)	35 (1.3)	89 (3.0)	96 (3.3)	114 (3.4)	111 (2.7)	206 (4.0)	192 (3.2)	144 (2.5)	78 (3.1)
Apixaban, n (%)	0 (0.0)	0 (0.0)	4 (0.1)	24 (0.8)	76 (2.6)	160 (4.8)	258 (6.3)	532 (10.3)	731 (12.1)	915 (15.8)	451 (17.9)
Warfarin, n (%)	257 (9.2)	242 (8.2)	252 (9.1)	218 (7.5)	193 (6.6)	162 (4.9)	162 (3.9)	141 (2.7)	98 (1.6)	61 (1.1)	18 (0.7)
None, n (%)	2513 (90.3)	2647 (89.7)	2443 (87.9)	2566 (87.7)	2545 (87.3)	2873 (86.5)	3579 (86.7)	4284 (82.7)	5024 (83.0)	4645 (80.4)	1965 (78.2)
Non-frail, N	22905	23987	23985	24247	24450	28410	31787	38625	42315	42418	18018
Dabigatran, n (%)	261 (1.1)	1592 (6.6)	1143 (4.8)	621 (2.6)	332 (1.4)	258 (0.9)	395 (1.2)	286 (0.7)	234 (0.6)	105 (0.2)	13 (0.1)
Rivaroxaban, n (%)	0 (0.0)	109 (0.5)	1001 (4.2)	1785 (7.4)	2122 (8.7)	1979 (7.0)	2179 (6.9)	3051 (7.9)	2774 (6.6)	2360 (5.6)	1005 (5.6)
Apixaban, n (%)	0 (0.0)	0 (0.0)	24 (0.1)	531 (2.2)	1541 (6.3)	3105 (10.9)	4400 (13.8)	6753 (17.5)	8900 (21.0)	10277 (24.2)	4755 (26.4)
Warfarin, n (%)	4633 (20.2)	3970 (16.6)	3875 (16.2)	3138 (12.9)	2725 (11.1)	2631 (9.3)	2272 (7.1)	2163 (5.6)	1273 (3.0)	969 (2.3)	402 (2.2)
None, n (%)	18011 (78.6)	18316 (76.4)	17942 (74.8)	18172 (74.9)	17730 (72.5)	20438 (71.9)	22541 (70.9)	26372 (68.3)	29135 (68.9)	28708 (67.7)	11845 (65.7)
Frail, N	3877	4288	4151	4297	4305	5033	5872	7393	8921	8619	3585
Dabigatran, n (%)	30 (0.8)	109 (2.5)	79 (1.9)	53 (1.2)	17 (0.4)	23 (0.5)	34 (0.6)	27 (0.4)	17 (0.2)	11 (0.1)	2 (0.1)
Rivaroxaban, n (%)	0 (0.0)	9 (0.2)	65 (1.6)	147 (3.4)	178 (4.1)	197 (3.9)	198 (3.4)	316 (4.3)	337 (3.8)	288 (3.3)	130 (3.6)
Apixaban, n (%)	0 (0.0)	0 (0.0)	5 (0.1)	44 (1.0)	141 (3.3)	304 (6.0)	524 (8.9)	917 (12.4)	1363 (15.3)	1678 (19.5)	773 (21.6)
Warfarin, n (%)	481 (12.4)	493 (11.5)	527 (12.7)	439 (10.2)	379 (8.8)	353 (7.0)	307 (5.2)	269 (3.6)	176 (2.0)	129 (1.5)	34 (0.9)
None, n (%)	3366 (86.8)	3677 (85.8)	3475 (83.7)	3614 (84.1)	3590 (83.4)	4156 (82.6)	4809 (81.9)	5865 (79.3)	7029 (78.8)	6513 (75.6)	2647 (73.8)
Stroke, N	2049	2078	2067	2160	2287	2755	3058	3808	4365	4173	1799
Dabigatran, n (%)	25 (1.2)	147 (7.1)	93 (4.5)	46 (2.1)	29 (1.3)	38 (1.4)	48 (1.6)	33 (0.9)	23 (0.5)	16 (0.4)	3 (0.2)
Rivaroxaban, n (%)	0 (0.0)	9 (0.4)	65 (3.1)	139 (6.4)	158 (6.9)	149 (5.4)	168 (5.5)	226 (5.9)	248 (5.7)	179 (4.3)	85 (4.7)
Apixaban, n (%)	0 (0.0)	0 (0.0)	3 (0.1)	38 (1.8)	164 (7.2)	327 (11.9)	470 (15.4)	711 (18.7)	1052 (24.1)	1097 (26.3)	490 (27.2)
Warfarin, n (%)	500 (24.4)	437 (21.0)	440 (21.3)	405 (18.8)	320 (14.0)	336 (12.2)	259 (8.5)	240 (6.3)	119 (2.7)	91 (2.2)	25 (1.4)
None, n (%)	1524 (74.4)	1485 (71.5)	1466 (70.9)	1532 (70.9)	1616 (70.7)	1905 (69.1)	2113 (69.1)	2598 (68.2)	2923 (67.0)	2790 (66.9)	1197 (66.5)
No stroke, N	24733	26197	26069	26384	26468	30688	34601	42210	46871	46864	19804
Dabigatran, n (%)	266 (1.1)	1554 (5.9)	1129 (4.3)	628 (2.4)	320 (1.2)	243 (0.8)	381 (1.1)	280 (0.7)	228 (0.5)	100 (0.2)	12 (0.1)
Rivaroxaban, n (%)	0 (0.0)	109 (0.4)	1001 (3.8)	1793 (6.8)	2142 (8.1)	2027 (6.6)	2209 (6.4)	3141 (7.4)	2863 (6.1)	2469 (5.3)	1050 (5.3)
Apixaban, n (%)	0 (0.0)	0 (0.0)	26 (0.1)	537 (2.0)	1518 (5.7)	3082 (10.0)	4454 (12.9)	6959 (16.5)	9211 (19.7)	10858 (23.2)	5038 (25.4)
Warfarin, n (%)	4614 (18.7)	4026 (15.4)	3962 (15.2)	3172 (12.0)	2784 (10.5)	2648 (8.6)	2320 (6.7)	2192 (5.2)	1330 (2.8)	1007 (2.1)	411 (2.1)
None, n (%)	19853 (80.3)	20508 (78.3)	19951 (76.5)	20254 (76.8)	19704 (74.4)	22689 (73.9)	25237 (72.9)	29639 (70.2)	33241 (70.9)	32431 (69.2)	13295 (67.1)
High CHA_2DS_2-VASc, N	22379	23475	23257	23580	23724	27510	31103	38258	43127	42934	18111
Dabigatran, n (%)	227 (1.0)	1356 (5.8)	989 (4.3)	550 (2.3)	280 (1.2)	220 (0.8)	336 (1.1)	261 (0.7)	201 (0.5)	98 (0.2)	11 (0.1)
Rivaroxaban, n (%)	0 (0.0)	89 (0.4)	839 (3.6)	1553 (6.6)	1837 (7.7)	1722 (6.3)	1852 (6.0)	2681 (7.0)	2466 (5.7)	2140 (5.0)	941 (5.2)

© 2022 Ko D et al. *JAMA Network Open.*

Characteristics	2010	2011	2012	2013	2014	2015	2016	2017	2018	2019	2020
Apixaban, n (%)	0 (0.0)	0 (0.0)	26 (0.1)	451 (1.9)	1361 (5.7)	2838 (10.3)	4090 (13.1)	6431 (16.8)	8708 (20.2)	10081 (23.5)	4662 (25.7)
Warfarin, n (%)	4311 (19.3)	3821 (16.3)	3798 (16.3)	3097 (13.1)	2580 (10.9)	2530 (9.2)	2150 (6.9)	2024 (5.3)	1186 (2.8)	916 (2.1)	331 (1.8)
None, n (%)	17841 (79.7)	18209 (77.6)	17605 (75.7)	17929 (76.0)	17666 (74.5)	20201 (73.4)	22675 (72.9)	26862 (70.2)	30568 (70.9)	29700 (69.2)	12169 (67.2)
Low CHA_2DS_2-VASc, N	4403	4800	4879	4964	5031	5933	6556	7760	8109	8103	3492
Dabigatran, n (%)	64 (1.5)	345 (7.2)	233 (4.8)	124 (2.5)	69 (1.4)	61 (1.0)	93 (1.4)	52 (0.7)	50 (0.6)	18 (0.2)	4 (0.1)
Rivaroxaban, n (%)	0 (0.0)	29 (0.6)	227 (4.7)	379 (7.6)	463 (9.2)	454 (7.7)	525 (8.0)	686 (8.8)	645 (8.0)	508 (6.3)	194 (5.6)
Apixaban, n (%)	0 (0.0)	0 (0.0)	3 (0.1)	124 (2.5)	321 (6.4)	571 (9.6)	834 (12.7)	1239 (16.0)	1555 (19.2)	1874 (23.1)	866 (24.8)
Warfarin, n (%)	803 (18.2)	642 (13.4)	604 (12.4)	480 (9.7)	524 (10.4)	454 (7.7)	429 (6.5)	408 (5.3)	263 (3.2)	182 (2.2)	105 (3.0)
None, n (%)	3536 (80.3)	3784 (78.8)	3812 (78.1)	3857 (77.7)	3654 (72.6)	4393 (74.0)	4675 (71.3)	5375 (69.3)	5596 (69.0)	5521 (68.1)	2323 (66.5)
High HAS-BLED[a], N	14216	15186	15156	15153	15134	17751	20320	25648	29074	28985	12173
Dabigatran, n (%)	149 (1.0)	940 (6.2)	638 (4.2)	356 (2.3)	158 (1.0)	154 (0.9)	244 (1.2)	175 (0.7)	146 (0.5)	65 (0.2)	9 (0.1)
Rivaroxaban, n (%)	0 (0.0)	69 (0.5)	577 (3.8)	1033 (6.8)	1204 (8.0)	1128 (6.4)	1281 (6.3)	1823 (7.1)	1716 (5.9)	1505 (5.2)	605 (5.0)
Apixaban, n (%)	0 (0.0)	0 (0.0)	24 (0.2)	276 (1.8)	882 (5.8)	1840 (10.4)	2729 (13.4)	4369 (17.0)	6162 (21.2)	7138 (24.6)	3240 (26.6)
Warfarin, n (%)	2842 (20.0)	2484 (16.4)	2467 (16.3)	2014 (13.3)	1580 (10.4)	1700 (9.6)	1434 (7.1)	1307 (5.1)	827 (2.8)	598 (2.1)	208 (1.7)
None, n (%)	11225 (79.0)	11693 (77.0)	11450 (75.5)	11474 (75.7)	11310 (74.7)	12929 (72.8)	14632 (72.0)	17975 (70.1)	20225 (69.6)	19679 (67.9)	8113 (66.6)
Low HAS-BLED[a], N	12566	13089	12980	13,391	13621	15692	17339	20370	22162	22052	9430
Dabigatran, n (%)	142 (1.1)	761 (5.8)	584 (4.5)	318 (2.4)	191 (1.4)	127 (0.8)	185 (1.1)	138 (0.7)	105 (0.5)	51 (0.2)	6 (0.1)
Rivaroxaban, n (%)	0 (0.0)	49 (0.4)	489 (3.8)	899 (6.7)	1096 (8.0)	1048 (6.7)	1096 (6.3)	1544 (7.6)	1395 (6.3)	1143 (5.2)	530 (5.6)
Apixaban, n (%)	0 (0.0)	0 (0.0)	5 (0.0)	299 (2.2)	800 (5.9)	1569 (10.0)	2195 (12.7)	3301 (16.2)	4101 (18.5)	4817 (21.8)	2288 (24.3)
Warfarin, n (%)	2272 (18.1)	1979 (15.1)	1935 (14.9)	1563 (11.7)	1524 (11.2)	1284 (8.2)	1145 (6.6)	1125 (5.5)	622 (2.8)	500 (2.3)	228 (2.4)
None, n (%)	10152 (80.8)	10300 (78.7)	9967 (76.8)	10312 (77.0)	10010 (73.5)	11665 (74.3)	12718 (73.3)	14262 (70.0)	15939 (71.9)	15542 (70.5)	6379 (67.6)
Anemia, N	6614	7165	7323	7517	7437	8558	9735	12078	14384	14144	5970
Dabigatran, n (%)	56 (0.8)	304 (4.2)	225 (3.1)	136 (1.8)	55 (0.7)	57 (0.7)	74 (0.8)	69 (0.6)	61 (0.4)	22 (0.2)	2 (0.0)
Rivaroxaban, n (%)	0 (0.0)	15 (0.2)	205 (2.8)	355 (4.7)	446 (6.0)	414 (4.8)	433 (4.4)	681 (5.6)	666 (4.6)	546 (3.9)	221 (3.7)
Apixaban, n (%)	0 (0.0)	0 (0.0)	11 (0.2)	92 (1.2)	317 (4.3)	682 (8.0)	976 (10.0)	1707 (14.1)	2538 (17.6)	3005 (21.2)	1427 (23.9)
Warfarin, n (%)	1076 (16.3)	1043 (14.6)	1038 (14.2)	906 (12.1)	723 (9.7)	727 (8.5)	650 (6.7)	586 (4.9)	402 (2.8)	278 (2.0)	103 (1.7)
None, %	5482 (82.9)	5803 (81.0)	5844 (79.8)	6028 (80.2)	5896 (79.3)	6678 (78.0)	7602 (78.1)	9036 (74.8)	10718 (74.5)	10293 (72.8)	4218 (70.7)
No Anemia, N	20168	21110	20813	21027	21318	24885	27924	33940	36852	36893	15633
Dabigatran, n (%)	235 (1.2)	1397 (6.6)	997 (4.8)	538 (2.6)	294 (1.4)	224 (0.9)	355 (1.3)	244 (0.7)	190 (0.5)	94 (0.3)	13 (0.1)
Rivaroxaban, n (%)	0 (0.0)	103 (0.5)	861 (4.1)	1577 (7.5)	1854 (8.7)	1762 (7.1)	1944 (7.0)	2686 (7.9)	2445 (6.6)	2102 (5.7)	914 (5.8)
Apixaban, n (%)	0 (0.0)	0 (0.0)	18 (0.1)	483 (2.3)	1365 (6.4)	2727 (11.0)	3948 (14.1)	5963 (17.6)	7725 (21.0)	8950 (24.3)	4101 (26.2)
Warfarin, n (%)	4038 (20.0)	3420 (16.2)	3364 (16.2)	2671 (12.7)	2381 (11.2)	2257 (9.1)	1929 (6.9)	1846 (5.4)	1047 (2.8)	820 (2.2)	333 (2.1)
None, n (%)	15895 (78.8)	16190 (76.7)	15573 (74.8)	15758 (74.9)	15424 (72.4)	17916 (72.0)	19748 (70.7)	23201 (68.4)	25446 (69.0)	24928 (67.6)	10274 (65.7)

© 2022 Ko D et al. *JAMA Network Open.*

Characteristics	2010	2011	2012	2013	2014	2015	2016	2017	2018	2019	2020
Chronic Kidney Disease, N	5284	6303	6608	,945	7275	9105	11710	15429	17829	18366	7905
Dabigatran, n (%)	44 (0.8)	253 (4.0)	193 (2.9)	118 (1.7)	58 (0.8)	64 (0.7)	105 (0.9)	109 (0.7)	79 (0.4)	41 (0.2)	4 (0.1)
Rivaroxaban, n (%)	0 (0.0)	16 (0.3)	190 (2.9)	372 (5.4)	441 (6.1)	465 (5.1)	591 (5.0)	946 (6.1)	924 (5.2)	884 (4.8)	396 (5.0)
Apixaban n (%)	0 (0.0)	0 (0.0)	4 (0.1)	101 (1.5)	389 (5.3)	869 (9.5)	1514 (12.9)	2599 (16.8)	3649 (20.5)	4472 (24.3)	2051 (25.9)
Warfarin, n (%)	964 (18.2)	1071 (17.0)	1130 (17.1)	1016 (14.6)	886 (12.2)	953 (10.5)	916 (7.8)	916 (5.9)	543 (3.0)	421 (2.3)	134 (1.7)
None, n (%)	4276 (80.9)	4963 (78.7)	5091 (77.0)	5338 (76.9)	5501 (75.6)	6754 (74.2)	8584 (73.3)	10860 (70.4)	12634 (70.9)	12548 (68.3)	5322 (67.3)
No Chronic Kidney Disease, N	21498	21,972	21528	21599	21480	24338	25949	30589	33407	32671	13698
Dabigatran, n (%)	247 (1.1)	1448 (6.6)	1029 (4.8)	556 (2.6)	291 (1.4)	217 (0.9)	324 (1.2)	204 (0.7)	172 (0.5)	75 (0.2)	11 (0.1)
Rivaroxaban, n (%)	0 (0.0)	102 (0.5)	876 (4.1)	1560 (7.2)	1859 (8.7)	1711 (7.0)	1786 (6.9)	2421 (7.9)	2187 (6.5)	1764 (5.4)	739 (5.4)
Apixaban, n (%)	0 (0.0)	0 (0.0)	25 (0.1)	474 (2.2)	1293 (6.0)	2540 (10.4)	3410 (13.1)	5071 (16.6)	6614 (19.8)	7483 (22.9)	3477 (25.4)
Warfarin, n (%)	4150 (19.3)	3392 (15.4)	3272 (15.2)	2561 (11.9)	2218 (10.3)	2031 (8.3)	1663 (6.4)	1516 (5.0)	906 (2.7)	677 (2.1)	302 (2.2)
None, n (%)	17101 (79.5)	17030 (77.5)	16326 (75.8)	16448 (76.2)	15819 (73.6)	17840 (73.2)	18766 (72.3)	21377 (69.9)	23530 (70.4)	22673 (69.4)	9170 (66.9)

Abbreviations: CHA_2DS_2-VASc score assigns 1 point for congestive heart failure, 1 point for hypertension, 2 points for age 75 years or older, 1 point for age 65 to 74 years,1 point for diabetes mellitus, 2 points for history of stroke or transient ischemic attack or systemic thromboembolism, 1 point for vascular disease including myocardial infarction or peripheral arterial disease, and 1 point for female sex; HAS-BLED score assigns 1 point each for hypertension, renal disease, liver disease, prior stroke, prior history of bleeding, age greater than 65, use of aspirin and other antiplatelets, and alcohol use disorder

[a]Excluded labile international normalized ratio component

© 2022 Ko D et al. *JAMA Network Open.*

eTable 2. OAC Mean Proportion of Days Covered and Nonadherence (Proportion of Days Covered < 80%) in OAC-Initiator Incident Atrial Fibrillation Cohort, 2010-2020

Characteristics	2010	2011	2012	2013	2014	2015	2016	2017	2018	2019	2020
All, N	4389	6263	6665	6743	7096	8703	10175	12876	15509	15874	8798
Median PDC [IQR]	77.6 [41.0, 96.4]	79.2 [41.0, 96.7]	82.0 [42.9, 97.3]	82.2 [49.2, 97.8]	82.0 [43.7, 96.7]	86.1 [57.1, 98.1]	86.9 [57.4, 97.8]	88.3 [57.4, 98.1]	87.2 [53.9, 98.1]	89.3 [57.4, 98.4]	90.2 [57.4, 98.6]
Non-adherent, n (%)	2290 (52.2)	3161 (50.5)	3169 (47.5)	3104 (46.0)	2175 (45.2)	3664 (42.1)	4221 (41.5)	5068 (39.4)	6442 (41.5)	6161 (38.8)	3434 (39.0)
Age 65-79 years, N	3221	4102	4078	4015	4377	5365	6370	8260	10092	10406	5746
Median PDC [IQR]	78.4 [41.9, 96.4]	78.7 [41.0, 96.4]	82.0 [41.0, 96.7]	82.0 [49.2, 97.5]	81.4 [41.0, 96.4]	85.8 [54.4, 98.1]	86.6 [55.2, 97.8]	88.0 [57.4, 97.8]	86.9 [52.2, 98.1]	89.3 [57.4, 98.4]	90.2 [57.4, 98.6]
Non-adherent, n (%)	1651 (51.3)	2090 (51.0)	1962 (48.1)	1875 (46.7)	2136 (48.8)	2292 (42.7)	2657 (41.7)	3284 (39.8)	4222 (41.8)	4116 (39.6)	2254 (39.2)
Age ≥80 years, N	1168	2161	2587	2728	2719	3338	3805	4616	5417	5468	3052
Median PDC [IQR]	73.8 [41.0, 96.2]	80.6 [42.7, 97.5]	82.0 [47.5, 98.4]	82.8 [49.2, 98.1]	82.2 [49.2, 97.3]	86.3 [57.4, 98.4]	86.9 [57.4, 97.5]	88.8 [59.4, 98.1]	87.4 [57.4, 98.4]	89.6 [58.1, 98.4]	89.9 [57.4, 98.8]
Non-adherent, n (%)	639 (54.7)	1071 (49.6)	1207 (46.7)	1229 (45.1)	1257 (46.2)	1372 (41.1)	1564 (41.1)	1784 (38.6)	2220 (41.0)	2045 (37.4)	1180 (38.7)
Women, N	2154	3142	3323	3332	3523	4372	5196	6499	7880	8070	4363
Median PDC [IQR]	78.1 [44.5, 97.0]	81.3 [45.3, 97.5]	82.0 [49.2, 97.8]	84.2 [49.2, 98.4]	82.5 [49.2, 97.3]	86.7 [57.4, 98.4]	87.3 [57.7, 97.8]	89.6 [63.4, 98.4]	88.5 [57.4, 98.4]	90.2 [62.3, 98.6]	91.0 [61.3, 99.2]
Non-adherent, n (%)	1106 (51.3)	1534 (48.8)	1552 (46.7)	1467 (44.0)	1619 (46.0)	1800 (41.2)	2084 (40.1)	2418 (37.2)	3095 (39.3)	2926 (36.3)	1580 (36.2)
Men, N	2235	3121	3342	3411	3573	4331	4979	6377	7629	7804	4435
Median PDC [IQR]	76.5 [41.0, 95.6]	77.6 [39.2, 95.9]	82.0 [41.0, 97.0]	82.0 [47.5, 97.0]	80.3 [41.0, 96.2]	85.2 [50.8, 97.8]	86.1 [51.9, 97.5]	86.6 [55.1, 97.8]	85.0 [49.2, 97.8]	87.4 [52.2, 98.4]	87.7 [51.9, 98.4]
Non-adherent, n (%)	1184 (53.0)	1627 (52.1)	1617 (48.4)	1637 (48.0)	1774 (49.7)	1864 (43.0)	2137 (42.9)	2650 (41.6)	3347 (43.9)	3235 (41.5)	1854 (41.8)
Asian, N	3475	4912	5207	5269	5586	6640	7827	9792	11481	11921	6580
Median PDC [IQR]	77.6 [42.3, 96.4]	79.2 [41.0, 97.0]	82.0 [42.9, 97.3]	83.1 [49.2, 97.8]	82.0 [48.6, 97.0]	86.9 [57.4, 98.4]	88.0 [57.4, 98.1]	89.3 [59.6, 98.3]	88.8 [57.4, 98.4]	90.2 [58.5, 98.6]	90.2 [57.7, 98.9]
Non-adherent, n (%)	1816 (52.3)	2478 (50.4)	2474 (47.5)	2371 (45.0)	2582 (46.2)	2734 (41.2)	3122 (39.9)	3722 (38.0)	4520 (39.4)	4401 (36.9)	2471 (37.6)
Black, N	310	475	505	471	438	528	753	1074	1558	1424	759
Median PDC [IQR]	73.8 [41.0, 95.2]	77.9 [32.8, 97.5]	82.0 [49.2, 96.6]	79.8 [41.7, 95.4]	75.3 [39.0, 94.9]	82.9 [49.2, 97.5]	84.2 [49.2, 96.4]	85.1 [52.4, 97.0]	82.0 [46.6, 96.2]	83.6 [53.6, 97.0]	84.7 [49.2, 98.1]
Non-adherent, n (%)	174 (56.1)	250 (52.6)	233 (46.1)	236 (50.1)	237 (54.1)	242 (45.8)	336 (44.6)	467 (43.5)	734 (47.1)	622 (43.7)	340 (44.8)
Hispanic, N	343	485	520	581	626	773	820	1190	1494	1496	812
Median PDC [IQR]	80.1 [41.0, 97.3]	79.8 46.6, 94.5]	82.0 [42.0, 98.4]	81.1 [49.2, 97.8]	73.8 [41.0, 95.1]	81.1 [45.6, 95.4]	76.8 [41.0, 95.3]	82.8 [49.2, 96.4]	80.3 [41.9, 96.2]	82.0 [49.2, 96.7]	83.4 [49.2, 97.8]
Non-adherent, n (%)	171 (49.9)	243 (50.1)	249 (47.9)	288 (49.6)	343 (54.8)	377 (48.8)	433 (52.8)	535 (45.0)	744 (49.8)	729 (48.7)	365 (45.0)
White, N	91	114	125	172	191	209	260	325	365	355	184
Median PDC [IQR]	86.6 [49.2, 98.9]	83.2 [34.4, 97.1]	82.0 [41.0, 97.5]	76.2 [40.9, 98.4]	82.0 [41.0, 97.0]	89.1 [58.9, 98.0]	86.9 [53.3, 98.3]	87.2 [57.4, 98.4]	82.0 [49.2, 98.4]	89.1 [49.2, 98.6]	88.1 [52.4, 98.4]

© 2022 Ko D et al. *JAMA Network Open.*

Characteristics	2010	2011	2012	2013	2014	2015	2016	2017	2018	2019	2020
Non-adherent, n (%)	38 (41.8)	52 (45.6)	60 (48.0)	88 (51.2)	94 (49.2)	86 (41.1)	105 (40.4)	135 (41.5)	175 (47.9)	138 (38.9)	75 (40.8)
Other, N	170	277	308	250	255	553	515	495	611	678	463
Median PDC [IQR]	72.5 [35.5, 94.9]	80.6 [35.1, 95.6]	80.3 [33.1, 97.7]	82.2 [49.2, 97.8]	73.8 [32.8, 96.2]	85.5 [57.8, 97.5]	86.1 [49.2, 97.5]	87.4 [60.7, 98.1]	83.6 [51.1, 98.1]	88.5 [57.4, 98.4]	90.4 [57.4, 98.4]
Non-adherent, n (%)	91 (53.5)	138 (49.8)	153 (49.7)	121 (48.4)	137 (53.7)	225 (40.7)	225 (43.7)	209 (42.2)	269 (44.0)	271 (40.0)	183 (39.5)
No Dementia, N	4112	5841	6196	6268	6613	8101	9365	11763	14044	14385	7888
Median PDC [IQR]	77.7 [42.1, 96.4]	79.5 [41.0, 96.7]	82.0 [43.4, 97.3]	82.2 [49.2, 97.8]	82.0 [44.5, 96.7]	86.3 [57.4, 98.1]	87.2 [57.4, 97.8]	88.5 [58.5, 98.1]	87.4 [55.5, 98.1]	89.6 [57.4, 98.4]	90.2 [57.4, 98.8]
Non-adherent, n (%)	2138 (52.0)	2936 (50.3)	2949 (47.6)	2890 (46.1)	3168 (47.9)	3388 (41.8)	386 (47.7)	4577 (38.9)	5773 (41.1)	5537 (38.5)	3032 (38.4)
Dementia, N	277	422	469	475	483	602	810	1113	1465	1489	910
Median PDC [IQR]	73.2 [33.6, 96.5]	74.5 [32.8, 98.4]	82.0 [41.2, 98.9]	82.5 [38.5, 98.1]	83.3 [41.0, 97.8]	83.2 [49.2, 97.5]	82.0 [48.3, 96.5]	85.0 [49.2, 97.8]	83.9 [47.6, 97.5]	86.3 [49.7, 98.1]	86.1 [49.2, 97.9]
Non-adherent, n (%)	152 (54.9)	225 (53.3)	220 (46.9)	214 (45.1)	225 (46.6)	276 (45.8)	3835 (41.0)	491 (44.1)	669 (45.7)	624 (41.9)	402 (44.2)
Non-frail, N	3696	5161	5545	5508	5895	7206	8466	10667	12466	12866	6930
Median PDC [IQR]	77.9 [42.9, 96.4]	79.8 [41.0, 96.4]	82.0 [46.4, 97.3]	82.5 [49.2, 97.8]	82.0 [45.1, 96.7]	86.9 [57.4, 98.1]	87.7 [57.4, 98.1]	89.1 [61.2, 98.1]	88.3 [57.4, 98.4]	90.2 [58.7, 98.6]	90.2 [57.4, 98.9]
Non-adherent, n (%)	1916 (51.8)	2586 (50.1)	2600 (46.9)	2528 (45.9)	2810 (47.7)	2961 (41.1)	3408 (40.3)	4061 (38.1)	5004 (40.1)	4815 (37.4)	2609 (37.6)
Frail, N	693	1102	1120	1235	1201	1497	1709	2209	3043	3008	1868
Median PDC [IQR]	75.1 [41.0, 95.7]	77.5 [39.3, 98.2]	79.0 [39.3, 98.1]	82.0 [44.6, 98.1]	82.0 [41.0, 97.5]	82.0 [43.2, 97.5]	82.0 [49.2, 96.2]	83.1 [48.9, 97.3]	82.0 [46.4, 96.7]	83.9 [49.2, 97.3]	85.6 [49.2, 98.1]
Non-adherent, n (%)	374 (54.0)	575 (52.2)	569 (50.8)	576 (46.6)	583 (48.5)	703 (47.0)	813 (47.6)	1007 (45.6)	1438 (47.3)	1346 (44.7)	825 (44.2)
Stroke, N	657	918	922	990	966	1142	1352	1559	2054	1960	1146
Median PDC [IQR]	82.2 [51.2, 97.3]	84.4 [57.9, 98.4]	88.0 [59.5, 98.9]	88.1 [60.3, 98.9]	88.7 [61.9, 98.9]	88.3 [60.8, 98.9]	89.3 [63.5, 98.4]	90.4 [65.6, 98.4]	89.1 [59.8, 98.4]	89.9 [62.3, 98.4]	92.6 [63.6, 99.5]
Non-adherent, n (%)	310 (47.2)	391 (42.6)	376 (40.8)	400 (40.4)	385 (39.9)	439 (38.4)	511 (37.8)	545 (35.0)	808 (39.3)	724 (36.9)	390 (34.0)
No stroke, N	3732	5345	5743	5753	6130	7561	8823	11317	13455	13914	7652
Median PDC [IQR]	76.2 [41.0, 96.2]	77.9 [39.3, 96.4]	81.4 [41.0, 97.0]	82.0 [47.5, 97.5]	80.9 [41.0, 96.3]	85.8 [55.6, 97.8]	86.3 [56.0, 97.5]	87.7 [57.4, 97.8]	86.6 [51.9, 98.1]	89.3 [57.4, 98.4]	89.3 [57.4, 98.6]
Non-adherent, n (%)	1980 (53.1)	2770 (51.8)	2793 (48.6)	2704 (47.0)	3008 (49.1)	3225 (42.7)	3710 (42.0)	4523 (40.0)	5634 (41.9)	5437 (39.1)	3044 (39.8)
High CHA_2DS_2-VASc, N	3917	5598	5952	6029	6257	7573	8859	11203	13644	13950	7745
Median PDC [IQR]	77.6 [42.3, 96.4]	79.5 [41.0, 96.8]	82.0 [45.5, 97.5]	82.1 [49.2, 97.8]	82.0 [45.1, 96.7]	85.5 [56.6, 98.1]	86.3 [57.4, 97.5]	87.7 [57.4, 97.8]	86.1 [52.2, 98.1]	88.8 [57.4, 98.4]	89.3 [57.4, 98.6]
Non-adherent, n (%)	2045 (52.2)	2816 (50.3)	2797 (47.0)	2781 (46.1)	2978 (47.6)	3227 (42.6)	3711 (41.9)	4469 (39.9)	5793 (42.5)	5490 (39.4)	3073 (39.7)
Low CHA_2DS_2-VASc, N	472	665	713	714	839	1130	1316	1673	1865	1924	1053
Median PDC [IQR]	76.8 [35.1, 97.2]	78.7 [32.8, 96.2]	77.9 [32.8, 95.6]	82.8 [49.0, 97.3]	80.3 [41.0, 96.7]	89.3 [57.4, 98.4]	90.2 [57.4, 98.4]	90.4 [65.6, 98.4]	91.3 [62.0, 98.4]	91.8 [65.6, 98.6]	92.6 [63.9, 99.5]
Non-adherent, n (%)	245 (51.9)	345 (51.9)	372 (52.2)	323 (45.2)	415 (49.5)	437 (38.7)	510 (38.8)	599 (35.8)	649 (34.8)	671 (34.9)	361 (34.3)

© 2022 Ko D et al. *JAMA Network Open.*

Characteristics	2010	2011	2012	2013	2014	2015	2016	2017	2018	2019	2020
High HAS-BLED[a], N	2795	4064	4247	4303	4415	5269	6409	8120	10214	10422	5845
Median PDC [IQR]	77.6 [41.0, 96.2]	78.7 [41.0, 97.0]	82.0 [41.0, 97.5]	82.0 [47.8, 97.5]	81.7 [41.0, 96.7]	84.7 [51.6, 97.8]	85.5 [54.4, 97.5]	87.2 [56.6, 97.8]	85.2 [49.2, 97.8]	88.0 [55.7, 98.4]	89.6 [57.4, 98.5]
Non-adherent, n (%)	1460 (52.2)	2073 (51.0)	2034 (47.9)	2032 (47.2)	2140 (48.5)	2310 (43.8)	2745 (42.8)	3342 (41.2)	4458 (43.6)	4202 (40.3)	2306 (39.5)
Low HAS-BLED[a], N	1594	2199	2418	2440	2681	3434	3766	4756	5295	5452	2953
Median PDC [IQR]	77.6 [45.1, 96.8]	80.3 [46.2, 96.4]	82.0 [49.2, 97.0]	83.7 [49.2, 98.1]	82.0 [49.2, 97.0]	88.0 [58.4, 98.4]	88.8 [58.7, 98.1]	89.9 [65.6, 98.4]	90.2 [59.6, 98.4]	90.7 [64.5, 98.6]	90.2 [57.4, 98.9]
Non-adherent, n (%)	830 (52.1)	1088 (49.5)	1135 (46.9)	1072 (43.9)	1253 (46.7)	1354 (39.4)	1476 (39.2)	1726 (36.3)	1984 (37.5)	1959 (35.9)	1128 (38.2)
Anemia, N	1107	1746	1793	1907	1858	2249	2631	3418	4498	4565	2682
Median PDC [IQR]	69.6 [31.1 93.2]	72.7 [29.7 95.9]	76.5 [32.8 96.3]	79.5 [36.9 97.0]	78.1 [35.5 96.2]	82.0 [47.6 97.5]	82.2 [49.2 96.7]	82.8 [48.6 97.3]	82.0 [44.0 96.9]	84.7 [49.2 97.8]	86.9 [49.2 98.4]
Non-adherent, n (%)	649 (58.6)	964 (55.2)	943 (52.6)	960 (50.3)	952 (51.2)	1051 (46.7)	1208 (45.9)	1557 (45.6)	2163 (48.1)	2032 (44.5)	1152 (43.0)
No Anemia, N	3282	4517	4872	4836	5238	6454	7544	9458	11011	11309	6116
Median PDC [IQR]	79.9 [48.6 97.0]	81.4 [49.2 97.0]	82.2 [49.2 97.5]	83.3 [50.0 97.8]	82.0 [49.2 96.7]	87.2 [57.4 98.4]	88.3 [57.4 98.1]	89.6 [63.9 98.4]	89.1 [57.4 98.4]	90.2 [62.0 98.6]	90.4 [58.5 98.9]
Non-adherent, n (%)	1641 (50.0)	2197 (48.6)	2226 (45.7)	960 (50.3)	2441 (46.6)	2613 (40.5)	3013 (39.9)	3511 (37.1)	4279 (38.9)	4129 (36.5)	2282 (37.3)
Chronic Kidney Disease, N	1013	1609	1817	1928	2032	2745	3658	4786	6245	6633	3804
Median PDC [IQR]	74.3 [41.0 95.8]	77.3 [36.2 97.0]	79.8 [41.0 97.3]	81.7 [41.0 97.5]	81.1 [41.0 97.3]	85.0 [50.0 97.8]	83.1 [50.1 97.0]	85.5 [54.4 97.5]	83.6 [49.2 97.4]	86.1 [52.3 98.1]	86.6 [51.5 98.4]
Non-adherent, n (%)	547 (54.0)	839 (52.1)	910 (50.1)	929 (48.2)	1002 (49.3)	1198 (43.6)	1664 (45.5)	2040 (42.6)	2827 (45.3)	2807 (42.3)	1604 (42.2)
No Chronic Kidney Disease, N	3376	4654	4848	4815	5064	5958	6517	8090	9264	9241	4994
Median PDC [IQR]	77.9 [42.9 96.7]	80.1 [41.0 96.7]	82.0 [45.9 97.3]	83.1 [49.2 97.8]	82.0 [45.1 96.4]	86.3 [57.4 98.4]	88.5 [57.4 98.1]	89.9 [62.3 98.4]	89.1 [57.4 98.4]	90.7 [60.5 98.6]	91.0 [59.6 99.2]
Non-adherent, n (%)	1743 (51.6)	2322 (49.9)	2259 (46.6)	2175 (45.2)	2391 (47.2)	2466 (41.4)	2557 (39.2)	3028 (37.4)	3615 (39.0)	3354 (36.3)	1830 (36.6)

Abbreviations: CHA_2DS_2-VASc score assigns 1 point for congestive heart failure, 1 point for hypertension, 2 points for age 75 years or older, 1 point for age 65 to 74 years,1 point for diabetes mellitus, 2 points for history of stroke or transient ischemic attack or systemic thromboembolism, 1 point for vascular disease including myocardial infarction or peripheral arterial disease, and 1 point for female sex; HAS-BLED score assigns 1 point each for hypertension, renal disease, liver disease, prior stroke, prior history of bleeding, age greater than 65, use of aspirin and other antiplatelets, and alcohol use disorder; PDC, proportion of days covered

[a]Excluded labile international normalized ratio component

© 2022 Ko D et al. *JAMA Network Open.*

eFigure. OAC Initiation and DOAC Uptake by Race and Additional Clinical Subgroups Over 2010-2020

Asian

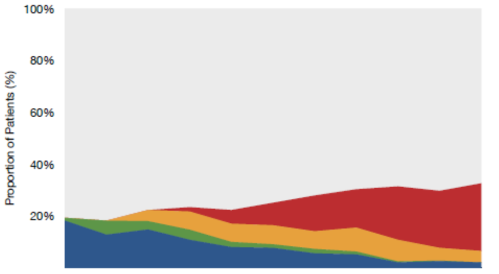

	2010	2011	2012	2013	2014	2015	2016	2017	2018	2019	2020
None	81%	82%	78%	77%	78%	75%	72%	70%	69%	70%	67%
Apixaban	0%	0%	0%	2%	5%	9%	14%	15%	21%	22%	26%
Rivaroxaban	0%	0%	4%	7%	7%	7%	7%	9%	8%	5%	4%
Dabigatran	1%	5%	3%	4%	2%	1%	2%	1%	0%	0%	0%
Warfarin	18%	13%	15%	11%	8%	8%	6%	5%	2%	3%	2%

Black

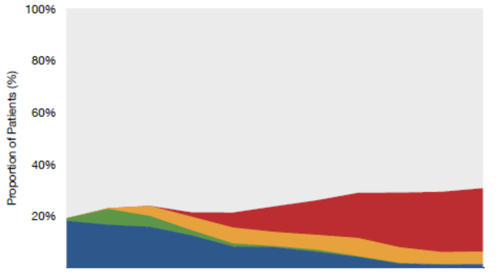

	2010	2011	2012	2013	2014	2015	2016	2017	2018	2019	2020
None	81%	77%	76%	79%	79%	76%	74%	71%	71%	71%	69%
Apixaban	0%	0%	0%	2%	6%	10%	13%	17%	21%	23%	25%
Rivaroxaban	0%	0%	4%	5%	6%	5%	6%	7%	6%	5%	5%
Dabigatran	1%	6%	4%	2%	1%	1%	1%	0%	0%	0%	0%
Warfarin	18%	17%	16%	13%	8%	8%	6%	4%	2%	1%	2%

Hispanic

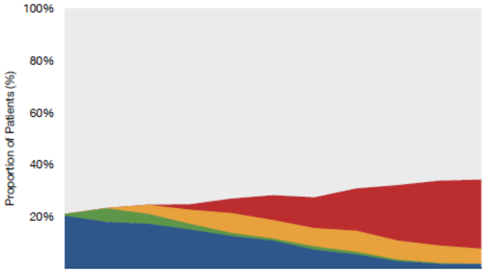

	2010	2011	2012	2013	2014	2015	2016	2017	2018	2019	2020
None	79%	77%	76%	75%	73%	72%	73%	69%	68%	66%	66%
Apixaban	0%	0%	0%	2%	6%	10%	12%	16%	21%	25%	27%
Rivaroxaban	0%	0%	4%	5%	8%	7%	7%	8%	7%	7%	6%
Dabigatran	1%	5%	4%	2%	1%	1%	1%	1%	1%	0%	0%
Warfarin	20%	18%	17%	15%	12%	11%	7%	5%	3%	2%	2%

White

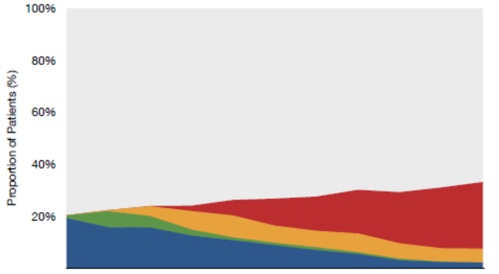

	2010	2011	2012	2013	2014	2015	2016	2017	2018	2019	2020
None	80%	78%	76%	76%	74%	73%	73%	70%	71%	69%	67%
Apixaban	0%	0%	0%	2%	6%	10%	13%	17%	20%	23%	26%
Rivaroxaban	0%	0%	4%	7%	8%	7%	6%	7%	6%	5%	5%
Dabigatran	1%	6%	4%	2%	1%	1%	1%	1%	1%	0%	0%
Warfarin	19%	16%	16%	13%	11%	9%	7%	5%	3%	2%	2%

Higher HAS-BLED

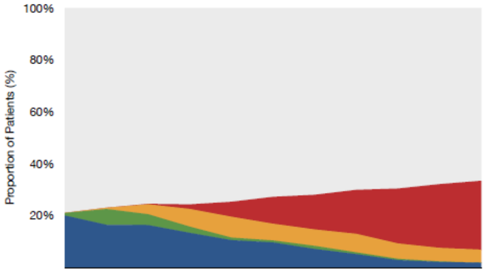

	2010	2011	2012	2013	2014	2015	2016	2017	2018	2019	2020
None	79%	77%	76%	76%	75%	73%	72%	70%	70%	68%	67%
Apixaban	0%	0%	0%	2%	6%	10%	13%	17%	21%	25%	27%
Rivaroxaban	0%	1%	4%	7%	8%	6%	6%	7%	6%	5%	5%
Dabigatran	1%	6%	4%	2%	1%	1%	1%	1%	1%	0%	0%
Warfarin	20%	16%	16%	13%	11%	10%	7%	5%	3%	2%	2%

Low HAS-BLED

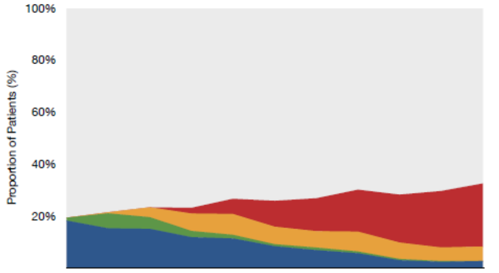

	2010	2011	2012	2013	2014	2015	2016	2017	2018	2019	2020
None	81%	79%	77%	77%	74%	74%	73%	70%	72%	71%	68%
Apixaban	0%	0%	0%	2%	6%	10%	13%	16%	19%	22%	24%
Rivaroxaban	0%	0%	4%	7%	8%	7%	6%	8%	6%	5%	6%
Dabigatran	1%	6%	5%	2%	1%	1%	1%	1%	1%	0%	0%
Warfarin	18%	15%	15%	12%	11%	8%	7%	6%	3%	2%	3%

Stroke

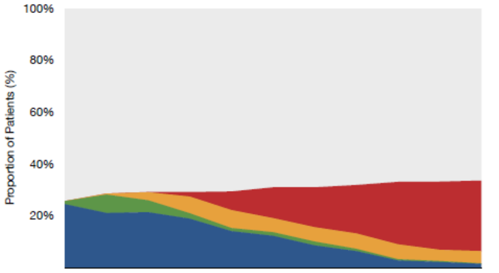

	2010	2011	2012	2013	2014	2015	2016	2017	2018	2019	2020
None	74%	72%	71%	71%	71%	69%	69%	68%	67%	67%	67%
Apixaban	0%	0%	0%	2%	7%	12%	15%	19%	24%	26%	27%
Rivaroxaban	0%	0%	3%	6%	7%	5%	6%	6%	6%	4%	5%
Dabigatran	1%	7%	5%	2%	1%	1%	2%	1%	1%	0%	0%
Warfarin	24%	21%	21%	19%	14%	12%	9%	6%	3%	2%	1%

No Stroke

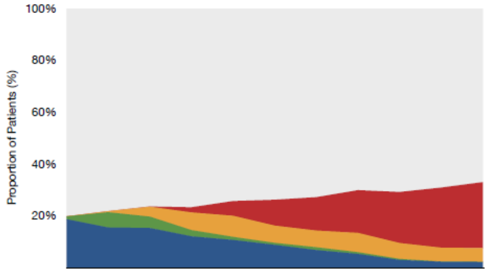

	2010	2011	2012	2013	2014	2015	2016	2017	2018	2019	2020
None	80%	78%	77%	77%	74%	74%	73%	70%	71%	69%	67%
Apixaban	0%	0%	0%	2%	6%	10%	13%	17%	20%	23%	25%
Rivaroxaban	0%	0%	4%	7%	8%	7%	6%	7%	6%	5%	5%
Dabigatran	1%	6%	4%	2%	1%	1%	1%	1%	0%	0%	0%
Warfarin	19%	15%	15%	12%	11%	9%	7%	5%	3%	2%	2%

© 2022 Ko D et al. *JAMA Network Open.*

eFigure. The stacked area plots depict the proportion of OAC use and DOAC update in the OAC-eligible incident AF cohort stratified by race, HAS-BLED score, and stroke. There was an increase in patients of Asian (OR for OAC initiation per year: 1.09, 95% CI 1.07-1.10, P < 0.001), Black (OR for OAC initiation per year: 1.06,95% CI 1.05-1.07, P < 0.001), and Other (OR for OAC initiation per year: 1.04, 95% CI 1.05-1.07, P < 0.001), White (OR for OAC initiation per year: 1.06, 95% CI 1.06-1.07, P < 0.001) race, and Hispanic ethnicity (OR for OAC initiation per year, 1.07, 95% CI 1.07-1.08, P < 0.001). There was an increase in OAC initiation in patients with lower (OR for OAC initiation per year, 1.06, 95% CI 1.06-1.07, P < 0.001) and higher (OR for OAC initiation per year, 1.07, 95% CI 1.06-1.07, P < 0.001) HAS-BLED and with (OR for OAC initiation per year, 1.03, 95% CI 1.02-1.04, P < 0.001) and without prior stroke (OR for OAC initiation per year, 1.07, 95% CI 1.06-1.07, P < 0.001).

© 2022 Ko D et al. *JAMA Network Open.*
